# Emission Properties of Wide InGaN/GaN Quantum Wells—Evidence for “Dark Charge” from Time-Resolved Photo- and Electroluminescence

**DOI:** 10.3390/ma19163501

**Published:** 2026-08-18

**Authors:** Witold Trzeciakowski, Artem Bercha, Mateusz Hajdel, Konrad Sakowski, Jens W. Tomm

**Affiliations:** 1 Institute of High Pressure Physics, Polish Academy of Sciences, 01-142 Warsaw, Poland; artem@unipress.waw.pl (A.B.); hajdel@unipress.waw.pl (M.H.); konrad@unipress.waw.pl (K.S.); 2 Institute of Applied Mathematics and Mechanics, University of Warsaw, Banacha 2, 02-097 Warsaw, Poland; 3 Max-Born-Institut, Max-Born-Str. 2A, D-12489 Berlin, Germany; tomm@mbi-berlin.de

**Keywords:** wide InGaN quantum wells, dark charge, time-resolved photoluminescence, time-resolved electroluminescence, InGaN LEDs and LDs, hot-carrier PL

## Abstract

InGaN/GaN quantum wells on polar substrates exhibit a pronounced quantum-confined Stark effect, which significantly limits their efficiency as light emitters. Surprisingly, this detrimental effect is significantly reduced when wider wells (above 10 nm) are used; their emission kinetics are the central focus of this work. A time range spanning nine orders of magnitude, from picoseconds to milliseconds, is explored through various experiments. This includes experiments on the optical visualization of slow decays of charge in the ground states (called “dark charge”) in the millisecond range, experiments on radiative recombination of excited states in the nanosecond range, and experiments on the relaxation of hot carriers in the picosecond range. All data are explained within the framework of qualitative and semi-quantitative models. The highly diverse kinetics of ground and excited states are due to the fact that the ground states of electrons and holes have negligible overlap and screen the built-in field, are optically inactive, and recombine nonradiatively in milliseconds. Meanwhile, when the field is screened, the excited states recombine radiatively in the picosecond/nanosecond ranges. The pulses of photo- and electroluminescence depend strongly on the excitation period. The application of negative-voltage pulses allows us to deplete the well of charge and generate short pulses of light.

## 1. Introduction

It was initially believed [1] that efficient InGaN/GaN emitters (LEDs and LDs) must have narrow (below 4 nm) quantum wells in the active area, since a strong electric field in the well (1–2 MV/cm) leads to a huge quantum-confined Stark effect (a reduction in the overlap of electron and hole states and a red shift of emission energy). This is a valid statement, but only for transitions involving ground states. Further studies of wide (8–25 nm)-quantum-well [QW] emitters have shown that the electric field may be screened by high carrier concentration, and efficient LEDs (Light Emitting Diodes) and LDs (Laser Diodes) have been demonstrated [2,3,4,5]. It is generally accepted that the emission from wide wells is dominated by excited-state transitions while charge in the ground state screens the built-in field very efficiently. Since this charge does not contribute to emission, it was named “dark charge” in [6] (in earlier papers [2,3,4,5], it was simply called “charge in the ground state”). The screening of electric fields increased interest in wide-QW structures, given that the detrimental effects of built-in fields are hard to eliminate. Alternatives such as growth on nonpolar substrates [7,8,9] resulted in lower structural quality, and high doping of the barriers increased nonradiative recombination [10]. Another advantage of wide wells is the stability of their emission wavelength with applied bias or varying excitation power.

Dark charge may appear in the well as a result of optical or electrical excitation. However, it seems that even without excitation, there is a finite concentration of dark charge in the well. The origins of dark charge may be due to the thermal generation of electrons and holes, followed by their separation by a strong electric field, thus preventing recombination. This would create some concentration of electrons and holes screening the field in the well, increasing nonradiative recombination until generation equals recombination. An extreme situation occurs when the voltage drop in the well exceeds the bandgap in the well. In this case, the Fermi level crosses both the edge of the conduction band and of the valence band so that electrons and holes appear at the two interfaces of the well.

Most earlier studies of wide-well structures were performed under CW operation [2,3,4,5]. In this review, we focus on time-dependent effects in photoluminescence (PL) and electroluminescence (EL), which reveal the unusual properties of wide wells due to dark charge. We summarize major experimental and theoretical results on transient properties of wide InGaN wells, starting from picoseconds up to milliseconds.

## 2. Materials and Methods

**Figure 1:**
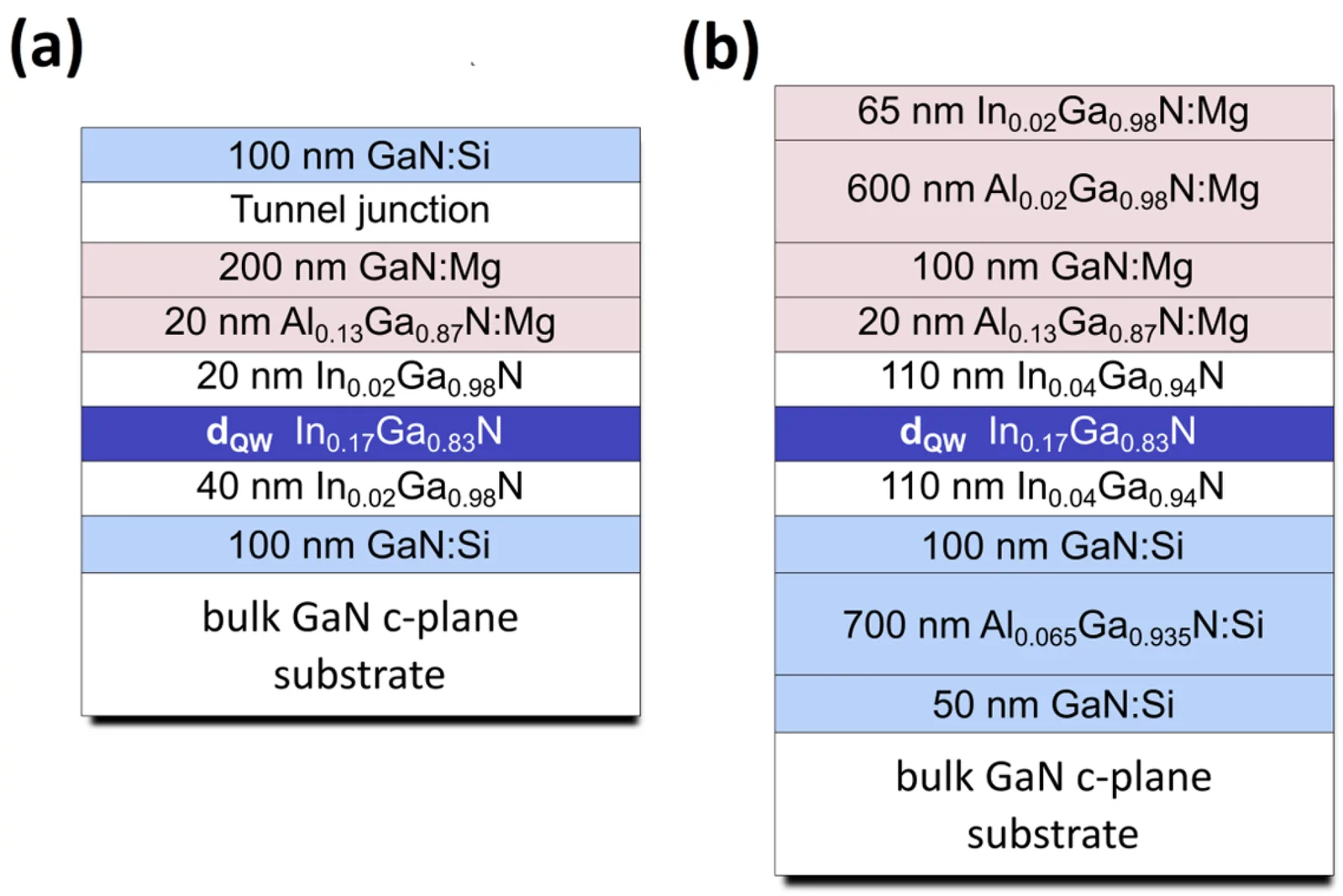
Structure of the typical wide-well LED sample (**a**) and LD sample (**b**). QW thicknesses were d_QW_ = 10.4, 15, or 25 nm (adapted with permission from [6] © Optica Publishing Group).

The studies that we review were all performed on MBE samples grown on bulk GaN substrates at the Institute of High Pressure Physics in Poland. We shall focus on well widths of 10.4 nm, 15 nm, and 25 nm. Reference samples with narrow well (2.6 nm wide) have also been grown for comparison. The composition of indium in the wells was 17%. In Figure 1, we show the typical structure of LEDs and LDs with a 10–25 nm well. The barriers in LEDs were 20–40 nm wide and contained 2% indium. In the case of laser structures, the 110 nm barriers contained 4% indium. For LEDs, the tunnel junction was grown above the structure, followed by a 110 nm n-type cap layer for better electrical contacts and current spreading. Metal contacts covered only a fraction of the LED surface, allowing for effective illumination or emission of light.

The experimental setups used for the time-dependent measurements were of three types.

For the picosecond/nanosecond resolution, the time-resolved PL system in the Max Born Institute was used with a femtosecond 400 nm frequency-doubled Spectra-Physics Tsunami Ti:sapphire laser used for excitation (80 MHz frequency), an Acton SP2300 0.3-m imaging monochromator, and a synchro-scan Hamamatsu C5680-21 streak camera with an S20 photocathode [11]. This means that the separation of laser pulses was only 12.5 ns. The resolution of the setup was better than 15 ps. The temperature of the sample was varied from 5 K up to 300 K.

The system for time-resolved electroluminescence at Chemnitz University of Technology used 80–800 ns current pulses for excitation with 20 kHz frequency (from a pulse generator Stanford Research Systems DG645, Sunnyvale, CA, USA). The emission passed by Princeton Instruments Acton SP2300 monochromator. A streak camera (Hamamatsu C10910) was used for detection of the electroluminescence spectrum [12,13]. The temporal resolution was below 1 ns. The temperature was set to 300 K.

In both studies with the streak camera, the period (frequency) of excitation was fixed.

The third type of time-resolved experiments was based on a photomultiplier (Hamamatsu R636-10) with photon counting (Stanford Research Systems, INC. SR400) having a 5 ns resolution. The emission was excited by pulses (from 10 μs up to 1 ms) of a 405 nm LD (Nichia NDV4B16) operated in CW mode and passed through an acousto-optic modulator (Brimrose TEM-85-10) or driven by a pulse generator (Siglend SDG 2040X). The detection of emitted light was accomplished by a gated photomultiplier attached to the monochromator (Spex 1000M by Horiba Jobin Yvon). The time gate of the photomultiplier was selected to be between 10 ns and 1 μs, and it was shifted with respect to the excitation pulse to measure the time evolution of the PL emission [6,14,15]. This method allowed us to vary all parameters: excitation power, duration, and period. Fast and slow decays (from 100 ns up to seconds) can be measured.

In each case, the excitation by 400–405 nm light was resonant, i.e., it generated electrons and holes only in the well.

Another experimental method used to determine (and reduce) the amount of charge in the quantum well was to apply negative-voltage pulses (NVPs) [13,14,15]. After the excitation pulse (by light or by current), the voltage applied to the diode was switched to a negative (reverse) value, reducing the field in the well and facilitating recombination (radiative and nonradiative). These pulses of negative voltage (from 1 μs up to 1 ms) were accompanied by short pulses of light (SPLs) of about 10–20 ns durations, as shown below.

## 3. Theoretical Background

**Figure 2:**
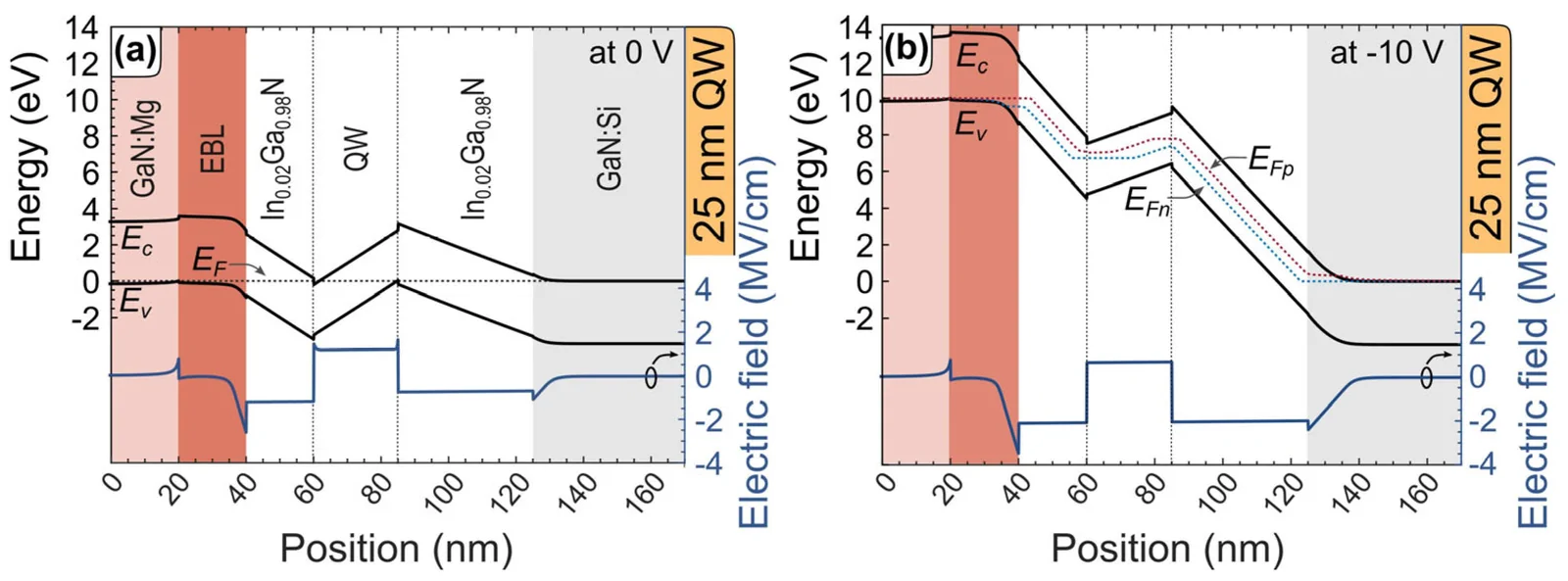
Potential profile of an LED with a 25 nm well at zero voltage (**a**) and at −10 V reverse voltage (**b**). Quasi-Fermi levels are marked by dotted lines; solid blue lines show the electric field profile. In (**a**), the Fermi level crosses the edges of the quantum well, which means that, at zero voltage, both electrons and holes are present in the well without any external excitation. This is not the case at −10 V (**b**) (reprinted with permission from [14]).

**Figure 3:**
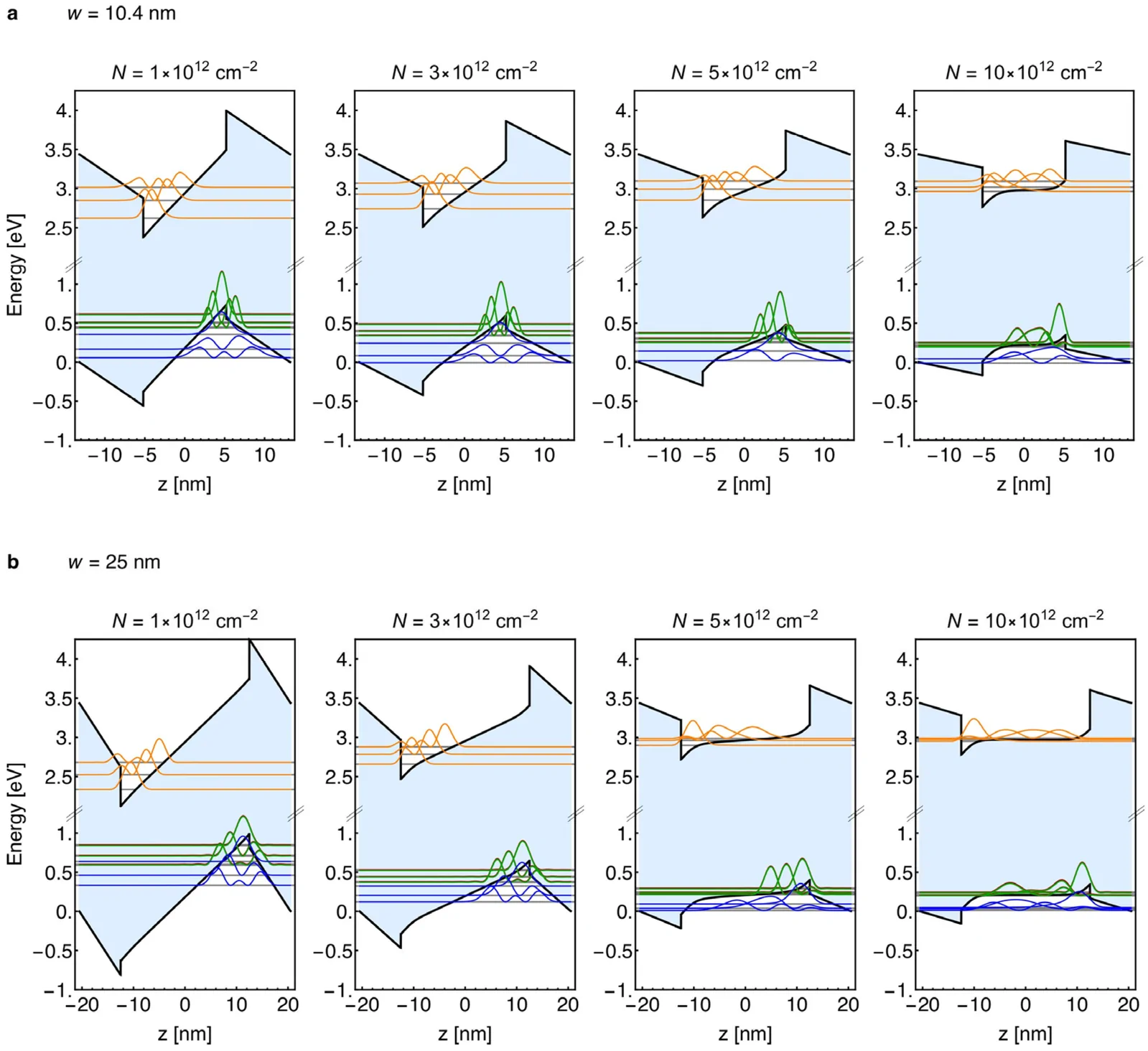
Potential, squared wavefunctions, and energy states for conduction and valence bands in (**a**) 10.4 nm and (**b**) 25 nm wide QWs, shown for different charge carrier densities (N). The orange lines show conduction band electron wavefunctions, whereas the heavy hole, light hole, and crystal-field split-off states are drawn in red, green, and blue, respectively. For clarity, only the three lowest states of each type are shown. Heavy- and light-hole wavefunctions are almost identical (green lines hide red lines), whereas the split-off states are barely involved in recombination (reprinted with permission from [12]).

In order to understand the peculiar properties of wide-well emitters, it is worth looking at the active layer of these structures as a function of external bias (Figure 2) and of carrier concentrations in the well (achieved by optical or electrical pumping; Figure 3).

We can see that a negative voltage reduces the field in the well and increases the probability of recombination.

A somewhat different reduction in the field can be achieved by increasing the carrier concentration in the well. The calculations in [12] were based on a 6 × 6 Hamiltonian for the valence states and the self-consistent solution of the Schrödinger and Poisson equations. The resulting potential profiles of the 10.4 nm and 25 nm In_0.17_Ga_0.83_N/GaN quantum wells are shown in Figure 3 as a function of carrier concentration, together with several eigenstates in the valence and conduction bands.

The wavefunctions plotted in Figure 3 demonstrate the transition from a 2D spectrum in a triangular well (at a low carrier concentration) to the almost-zero-field 3D spectrum (at a high carrier concentration), as discussed in [12]. This simulation also demonstrates that the ground states in the well remain spatially separated even when the field is zero in most of the well. This is why they do not contribute to emission but screen the built-in field effectively.

**Figure 4:**
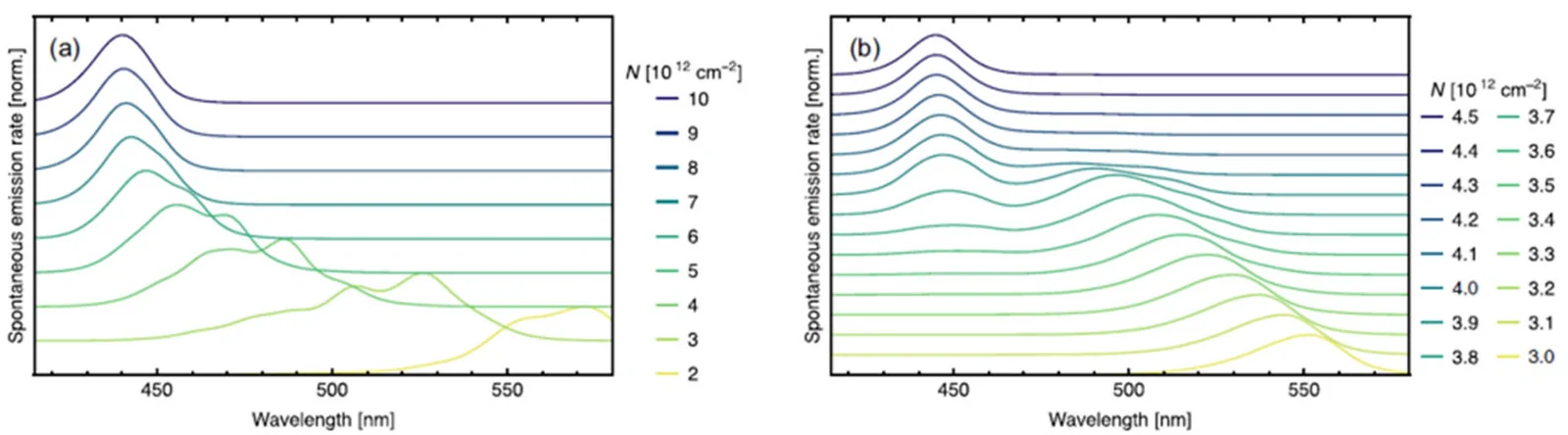
Normalized spontaneous emission spectra calculated for (**a**) 10.4 nm and (**b**) 25 nm wide QWs at different carrier densities (reprinted with permission from [12]).

Based on this model, the emission spectra were calculated, also as a function of carrier concentration in the well (Figure 4).

These spectra show huge shifts in the emission lines from yellow to blue. However, they are normalized, and the normalization factors are not shown, so we do not know the intensity of these spectra. In fact, such shifts have never been experimentally observed in wide wells. On the contrary, we already mentioned the stability of the emission energy as a function of excitation power. This stability, however, is seen only at high carrier concentrations. The emission at low concentrations is simply not observable.

**Figure 5:**
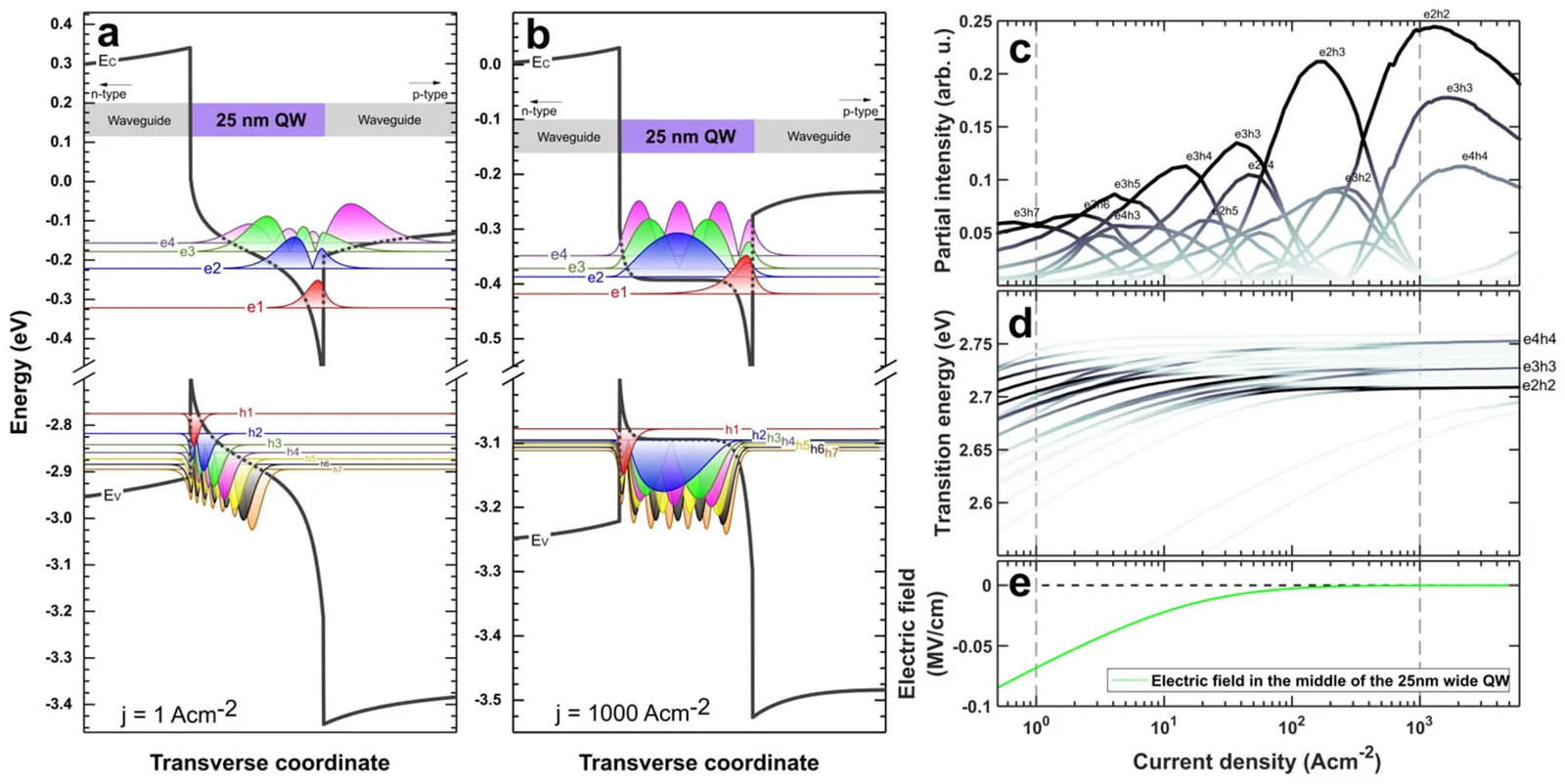
Simulated band structure of a 25 nm wide QW at (**a**) *j* = 1 A cm^−2^ and (**b**) *j* = 1000 A cm^−2^ with wavefunctions of the first seven heavy hole levels and four electron levels. Energy values are indicated by base lines, and wavefunctions are normalized and scaled for better visibility. (**c**) Calculated dependence of partial intensity of the most important transitions on current density. (**d**) Dependence of transition energies for transitions presented in (**c**) on current density. The color scale represents the contribution to total intensity and is identical to that in panel (**c**). (**e**) Dependence of the electric field in the middle of the 25 nm wide QW on current density. The dashed vertical lines in panels (**c**–**e**) indicate the excitation levels, which correspond to band structures shown in panels (**a**,**b**) (reprinted with permission from [16]).

Another calculation interpreting the stability of emission wavelength (with current) in wide wells was presented in [16], where it was shown that the stable emission energy starts at a nonzero electric field in the well, but different pairs of excited states contribute to emission, keeping the average energy constant (Figure 5), even when the field varies.

The intensity of the interband transitions depends on the overlap of the electron and hole wavefunctions, and on the occupation of the two states involved. An important factor is the sensitivity of the detector, i.e., the observable threshold intensity. In order to compare the two simulations, we calculated the carrier concentrations as a function of current density (in the experimentally observable range from 1 up to 5000 A/cm^2^) using the simulation described in [16]. We obtained the values given in Table 1.

Meanwhile, the range of carrier densities in [12] was from 10^12^ up to 10^13^/cm^2^, i.e., an order of magnitude lower. Moreover, the piezoelectric fields were assumed to be three times stronger in [16], increased additionally due to the applied external voltage of around 3 V (which was absent in [12]). In the range around 10^13^/cm^2^, both calculations show a stable emission wavelength (independent of current or concentration), which is characteristic of wide-well structures.

## 4. Experimental Results

### 4.1. Emission from Wide Wells in the Picosecond/Nanosecond Range

**Figure 6:**
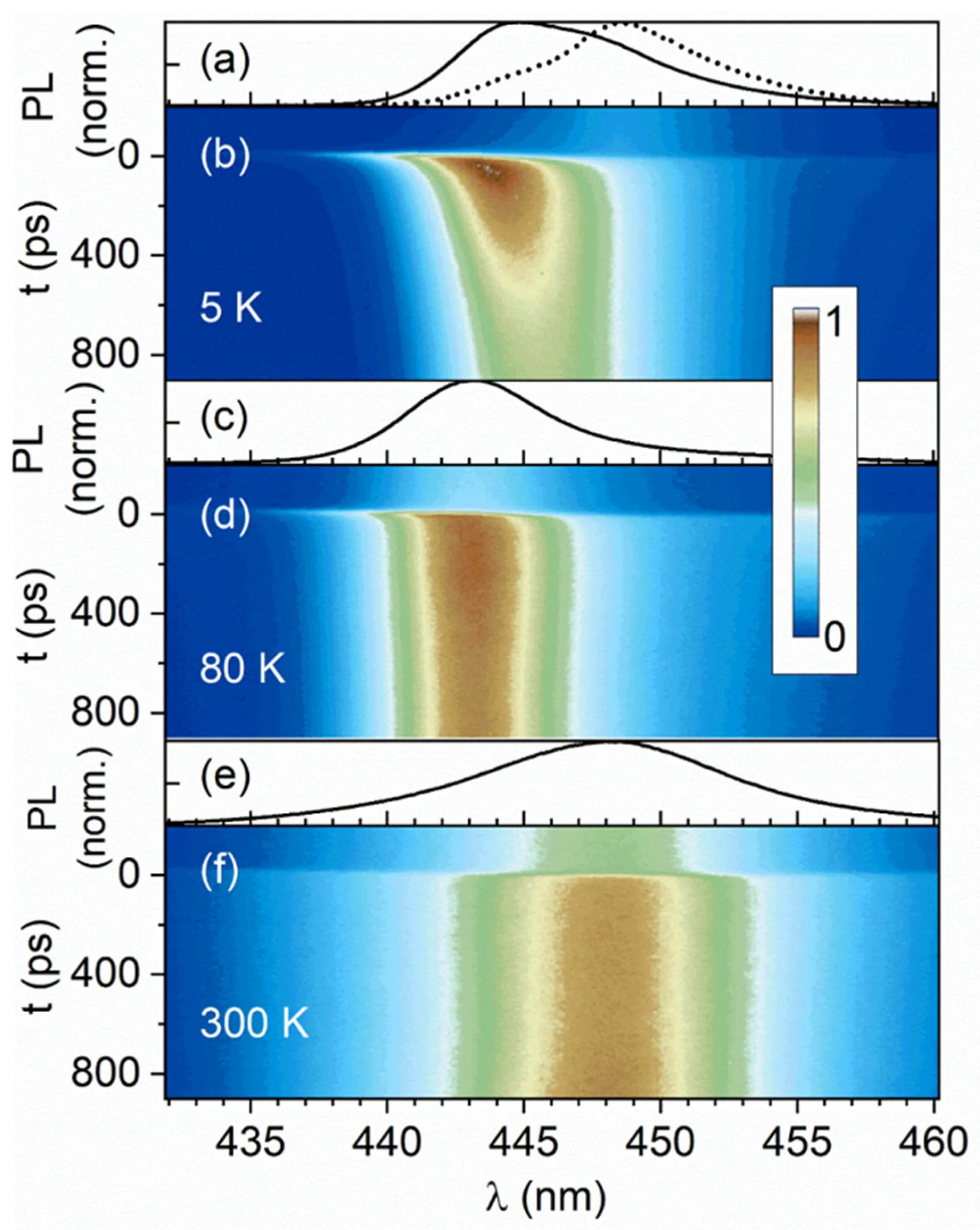
(**a**–**f**) Normalized transient PL spectra from the 25 nm QW sample recorded at (**a**,**b**) T = 5 K, (**c**,**d**) T = 80 K, and (**e**,**f**) T = 300 K for an excitation of 10^11^ carrier pairs per pulse and cm^2^ with 400 nm photons. While (**a**,**c**,**e**) show spectra time-averaged over the entire 12.5 ns period between two excitation pulses as full lines, (**b**,**d**,**f**) display maps of the spectral–temporal behavior during only 1 ns, including t = 0 (position of the excitation pulse that is not shown) and the initial 850 ps of the PL decay. The dotted spectrum in (**a**) is time-averaged in the time interval of −200 to −100 ps, i.e., the PL just before the pulse (reprinted with permission from [11]).

In Ref. [11], the time-resolved PL spectra from the 25 nm well were measured from 5 K up to 300 K (Figure 6).

The above spectra cover an approximately one-nanosecond range, and the signal below t = 0 comes from the previous pulse (12.5 ns before the current pulse).

**Figure 7:**
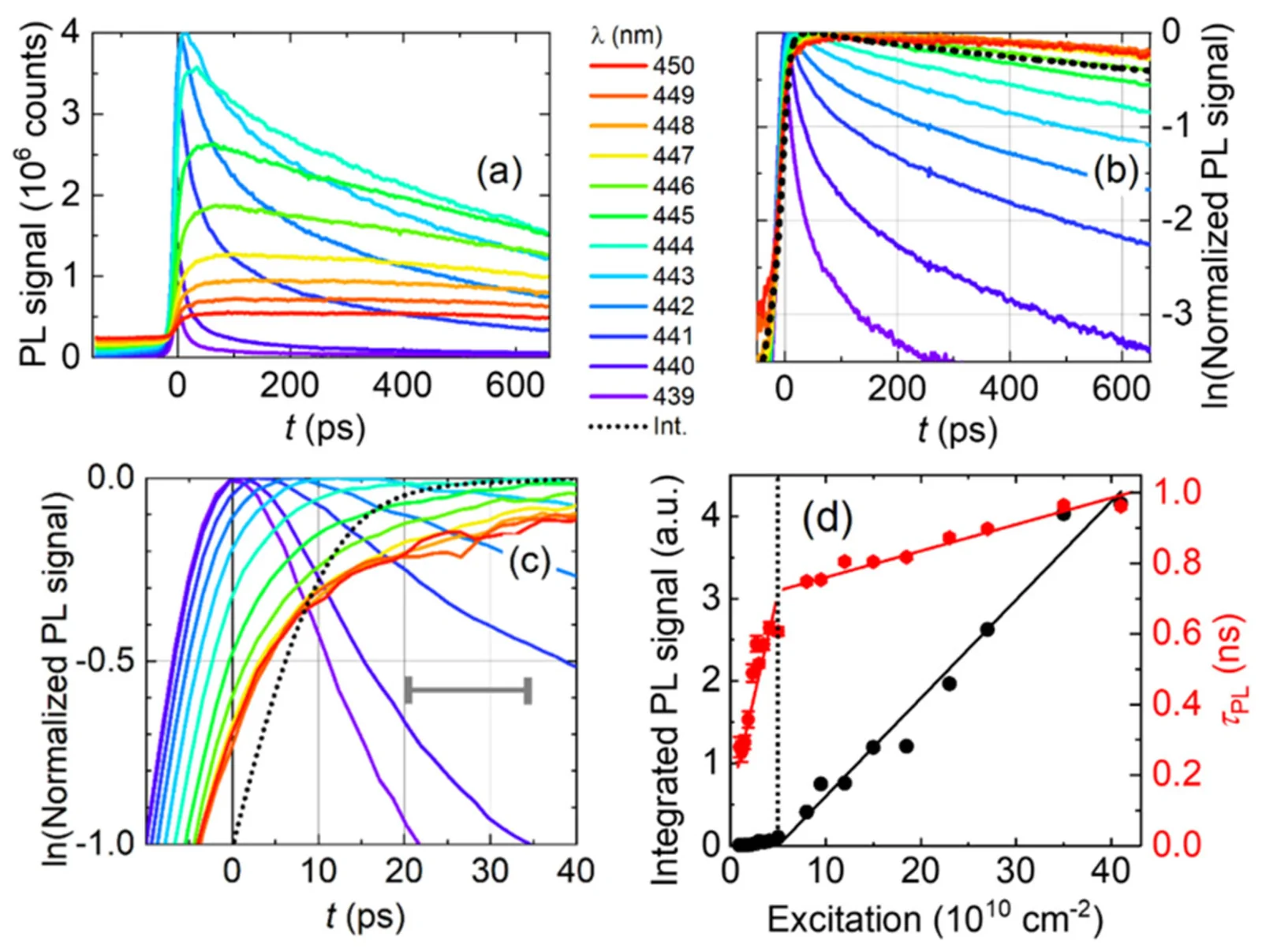
(**a**) PL transients at different wavelengths at T = 5 K and an excitation of 10^11^ carrier pairs per pulse and cm^−2^ taken as vertical cuts from PL maps as shown in Figure 6b. (**b**) The same dataset but normalized and in a logarithmic plot. The dotted line represents the wavelength-integrated transient. (**c**) The same as (**b**) on a shorter timescale. The gray bar visualizes the time resolution. The color codes of the temperatures in the top panel apply to (**a**–**c**). (**d**) Power dependences of the PL signal averaged in time over the full 12.5 ns period between two excitation pulses (black) and wavelength-integrated τ_PL_ taken in the 200–650 ps time window (red) at T = 5 K. The dotted line marks the threshold excitation (reprinted with permission from [11]).

Some analysis of the wavelength-resolved 5 K spectra is shown in Figure 7. The main conclusions of this data can be summarized as follows:

(a) Wide-QW structures show almost exponential ns-kinetics (Figure 7b, dotted line) and behave as if they are dominated by radiative recombination (since decay times increase with temperature).
(b) At low excitation densities, the dark charge forms first, and PL from excited states appears when the field is screened. This leads to the appearance of a threshold of the PL (and *τ*_PL_) (Figure 7d).

(c) A very broad spectrum of time constants (from ps to ns) is found when PL is spectrally resolved. At low temperatures, PL decays as fast as 16 ps were found. This PL is assigned to hot-carrier PL within the bulk-like quasi-continuum of states. At low temperatures, the carriers photogenerated high in the bands recombine mainly through interactions with acoustic phonons, which is a fairly slow process. This is why interband transitions at higher energies are possible. At higher temperatures, the tails of the Fermi distribution allow for recombination with the emission of optical phonons, which is very fast. Therefore, the “phonon bottleneck” [17] seems to be the reason for the observation of higher-energy transitions at low temperature.

### 4.2. Electroluminescence from Wide Wells in the Nanosecond Range

**Figure 8:**
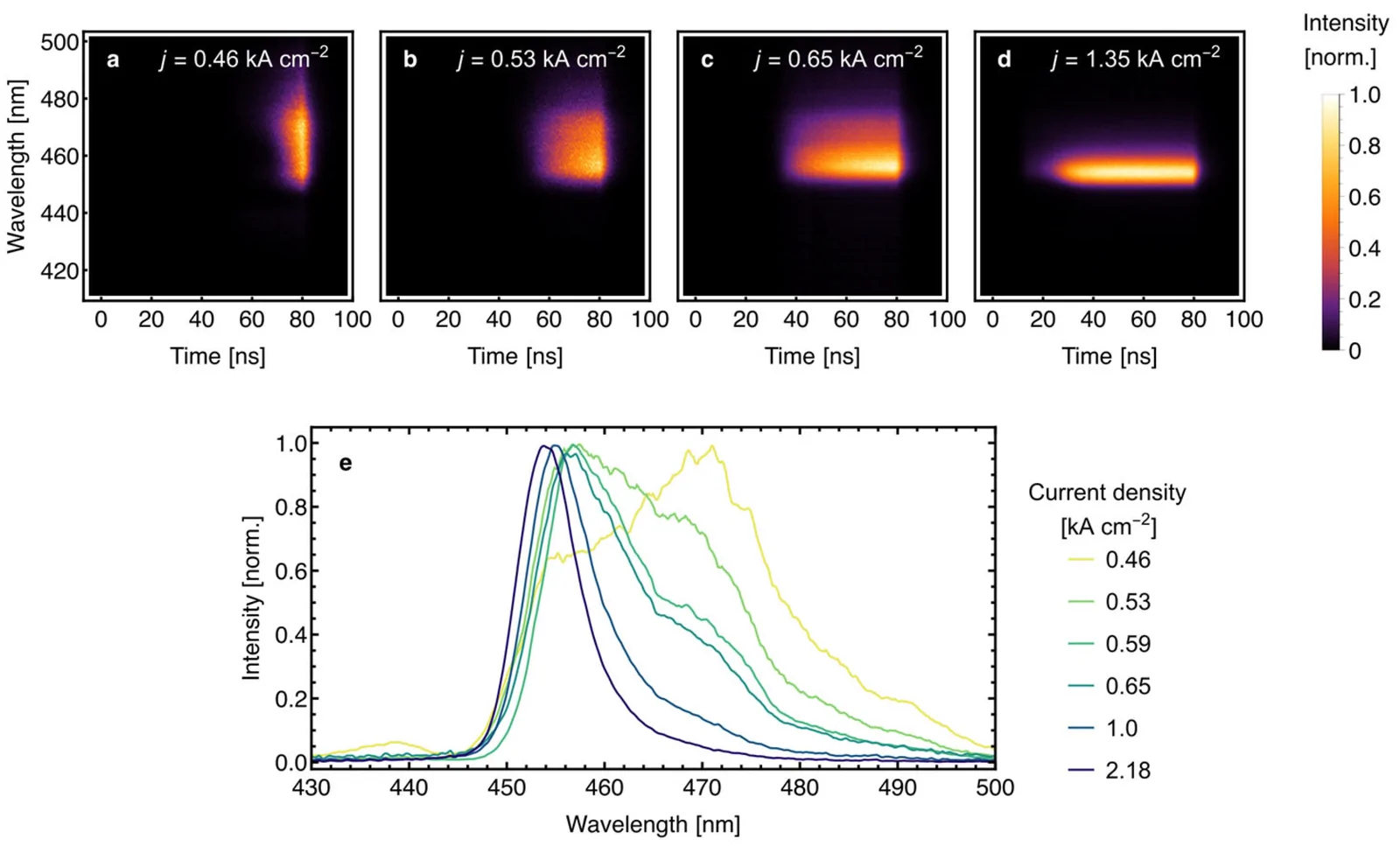
Streak camera measurements on the 10.4 nm thick QW. (**a**–**d**) Spectral–temporal dynamics and (**e**) time-integrated normalized spectra for different pulse currents. The 80 ns long driving pulse starts at t = 0 ns. Images (**a**,**b**) were measured using photon counting and (**c**,**d**) in analog integration mode (reprinted with permission from [12]).

In Ref. [12], the electroluminescence emission from wide wells (10.4 and 25 nm) was studied using 80 ns current pulses of varying current density *j* (Figure 8).

Similar results were obtained for the 25 nm well. The emission starts with some delay with respect to the excitation pulse. This delay decreases with increasing current density, which is consistent with the requirement of a sufficient concentration of dark charge filling the ground states and screening the built-in field. For low current densities, the emission shows a low-intensity (50 times lower) peak at 470 nm, and for higher densities, it saturates at 455 nm. In CW experiments, this longer wavelength emission at low excitation has not been observed.

**Figure 9:**
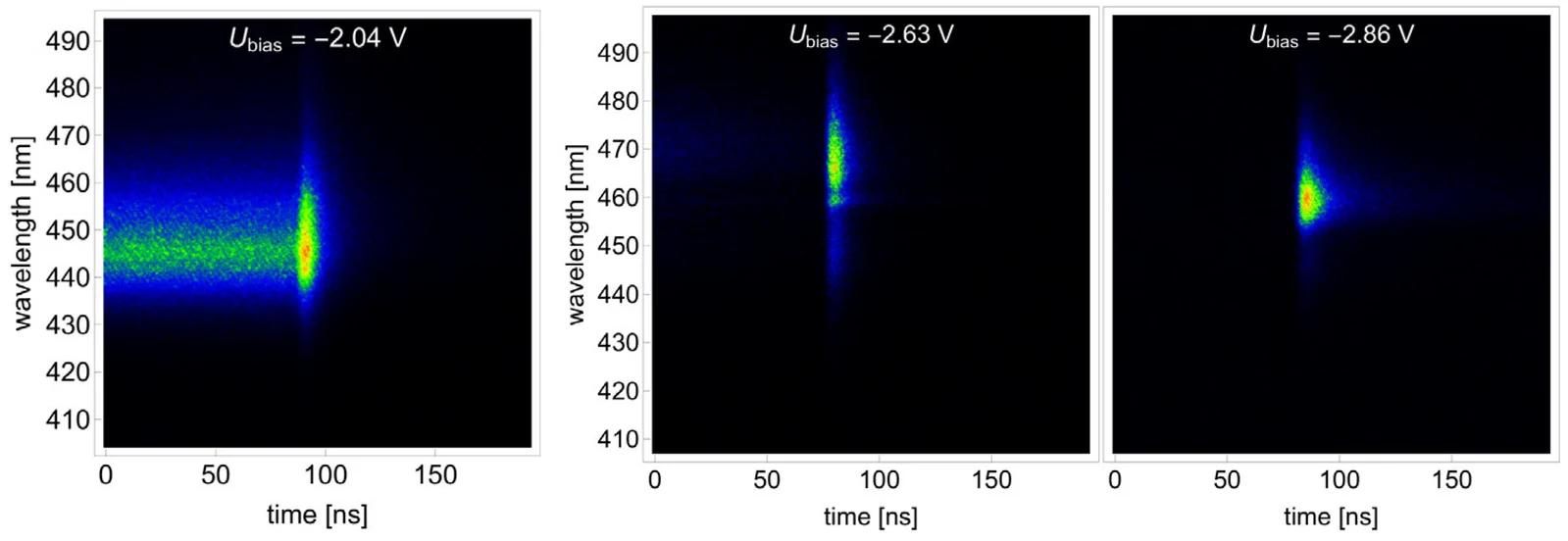
Streak camera images for the measurement on a laser diode with a 10.4 nm wide QW (**left**) and a 25 nm wide QW (**right**). The values of negative bias are shown (reprinted with permission from [13]).

Using the same experimental setup, it was shown in [13] that bright emission occurs at the trailing edge of the excitation pulse if the voltage is rapidly switched from a positive (3.3 V) to a negative (reverse) value (Figure 9). In this case, the excitation pulse (at 3.3 V) was long (800 ns) so as to obtain a stationary carrier concentration in the well.

**Figure 10:**
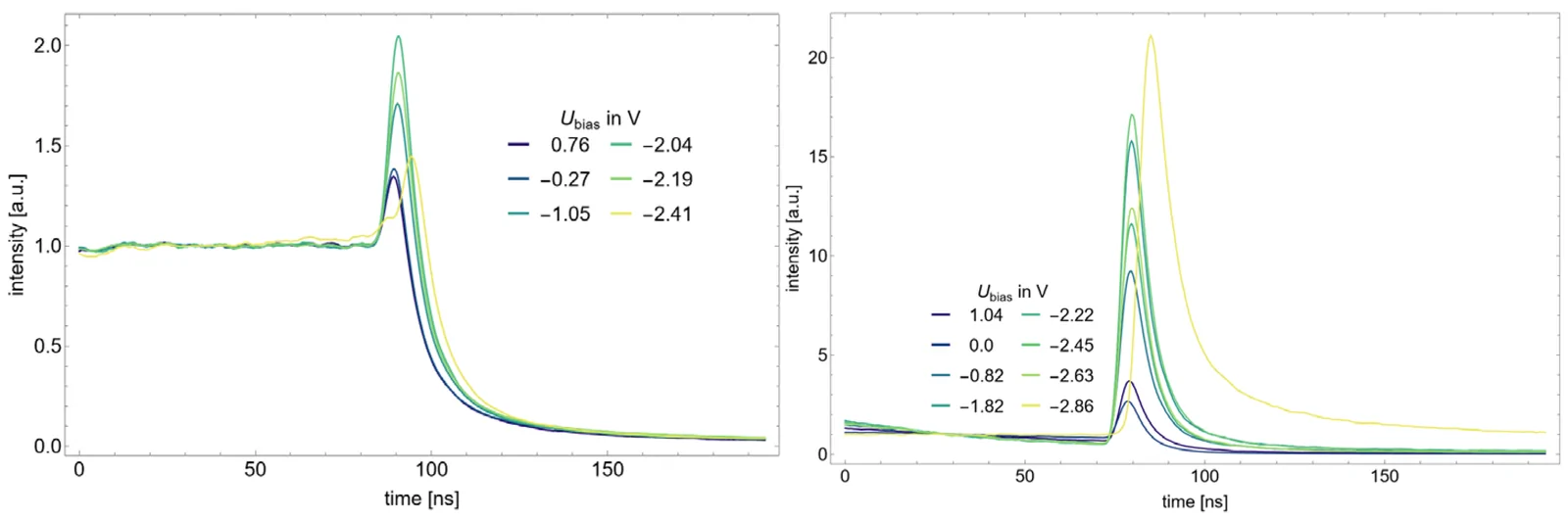
Time dependence of EL intensity for several values of applied reverse bias for 10.4 nm (**left**) and 25 nm wide QW (**right**). The values of negative bias are shown (reprinted with permission from [13]).

The figure shows the last 100 ns of the pulse. At the end of the pulse, we can see a strong increase in emission for the 10.4 nm well, and the appearance of a short pulse of light in the case of the 25 nm well. This effect has also been observed and studied in more detail in time-resolved studies in the microsecond range, which will be described later. In Figure 10, the emission intensity vs. time is shown. These short pulses appear due to the reduction in the electric field by reverse bias, so that the charge trapped in the well recombines through excited states (due to rapid transfer of dark charge to excited states).

These short pulses were observed in laser structures, so there was a discussion in [15] about whether they could be made strong enough to generate lasing.

We shall now review some time-dependent studies in the microsecond (and above) range showing very slow decay and “regeneration” effects of dark charge.

### 4.3. PL Emission from Wide Wells in the Microsecond Range

**Figure 11:**
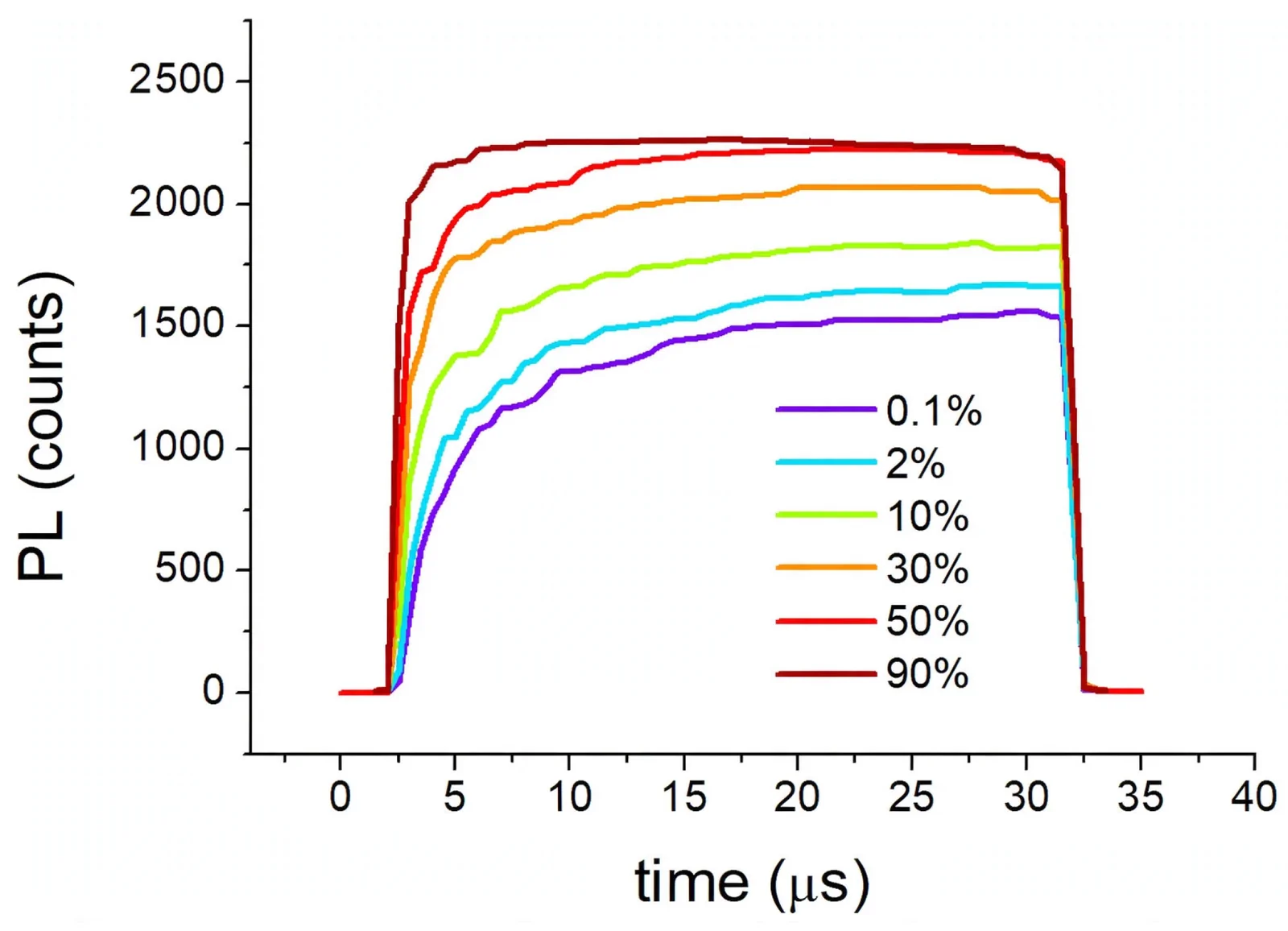
The effect of different spacing (fill factor) of 30 μs pulses (15 nm well, spacing from 3.3 μs to 29,970 μs) for excitation power of 7 mW (reprinted with permission from [6] © Optica Publishing Group).

The evidence for the existence of dark charge was supplied by the study of PL emission excited by 10–30 μs pulses of 405 nm LD in 15 and 25 nm wells [6]. It was found that the shape and intensity of PL emission were dependent on the temporal separation (fill factor) of exciting pulses (Figure 11). In narrow quantum wells, the PL emission rises and decays in nanoseconds, and the separation of the pulses does not matter.

In [6], a simple model of PL emission from two coupled states explained some of the observed features but also predicted slow decay of PL pulses, contrary to the experiment.

**Figure 12:**
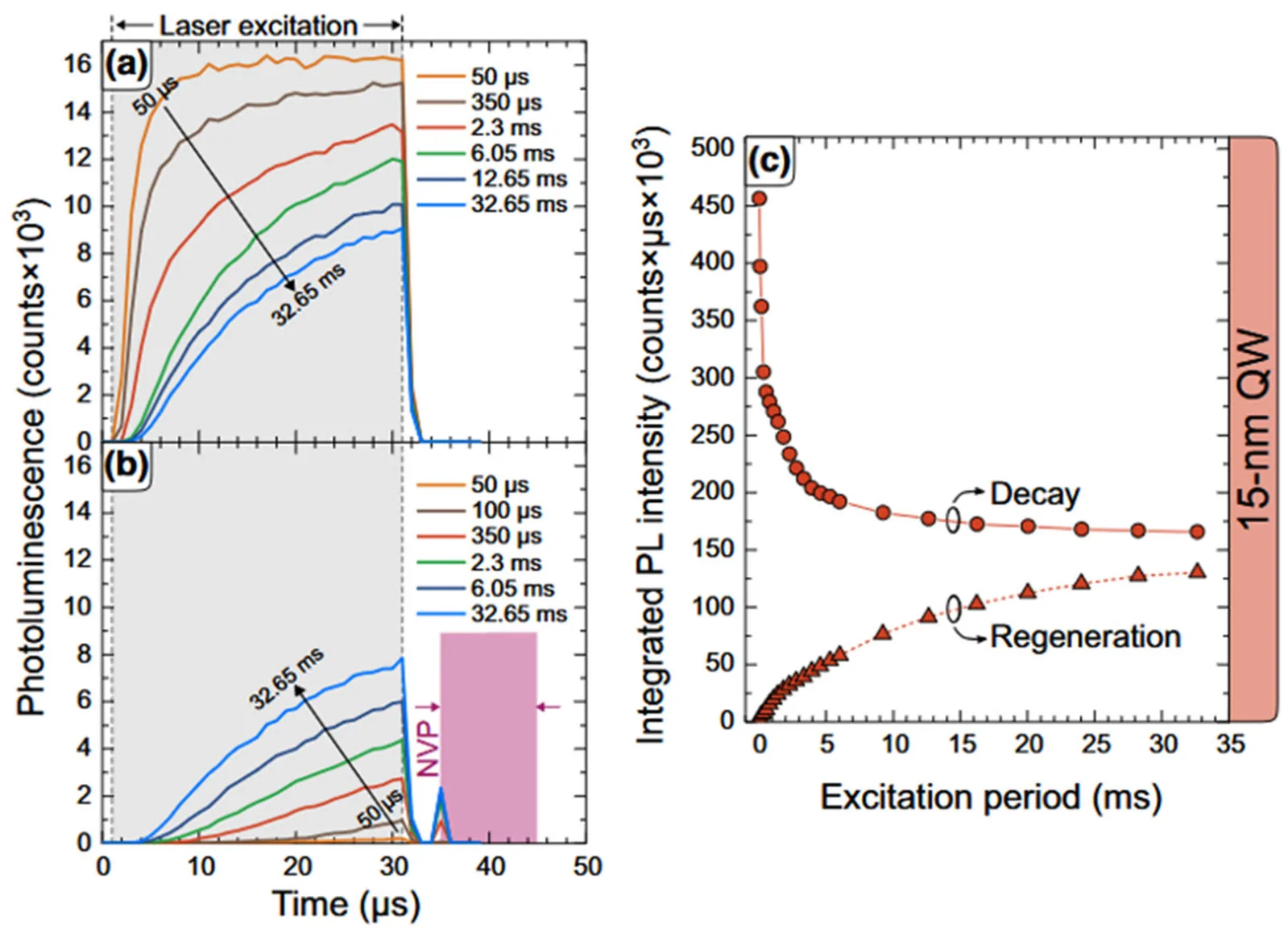
PL pulses from LED with 15 nm well (excited by 3 mW 30 μs pulses of 405 nm LD) as a function of excitation period (from 50 μs to 32.65 ms) (**a**) without the NVP, (**b**) with the NVP (−10 V, 10 μs duration) applied 3 μs after the laser pulse; (**c**) integrated intensity of PL shown in (**a**,**b**) (reprinted with permission from [14]).

A more detailed study of PL pulses as a function of their separation was performed in [14]. In addition, negative-voltage pulses (NVPs) were applied after the laser pulses, reducing the dark charge concentration. The periodic excitation without NVPs revealed a monotonic decrease in PL pulses when their separation was increased, up to about 30 ms. Above 30 ms, the intensity and shape of the PL pulses stabilized (Figure 12a). This reflects slow decay of dark charge.

When the NVPs were applied 3 μs after the laser pulse (−10 V for 10 μs), the intensity of PL pulses increased with increasing separation of excitation pulses (Figure 12b). This effect (called “regeneration of dark charge”) demonstrated that after depleting the well from dark charge, its concentration slowly increases with time and saturates at around 30 ms. This implies that there is some equilibrium population of “dark charge” in a wide well. This charge is present in the QW without any excitation. If we reduce the population of “dark charge” (by NVP) below this equilibrium value, it will increase (or “regenerate”) towards the equilibrium population within tens of milliseconds. In Figure 12c, the integral of PL pulses is shown as a function of excitation period. It seems that the decay curve and the regeneration curve tend toward the same limit. The initial changes (up to about 1 ms) are much faster than those above 1 ms.

**Figure 13:**
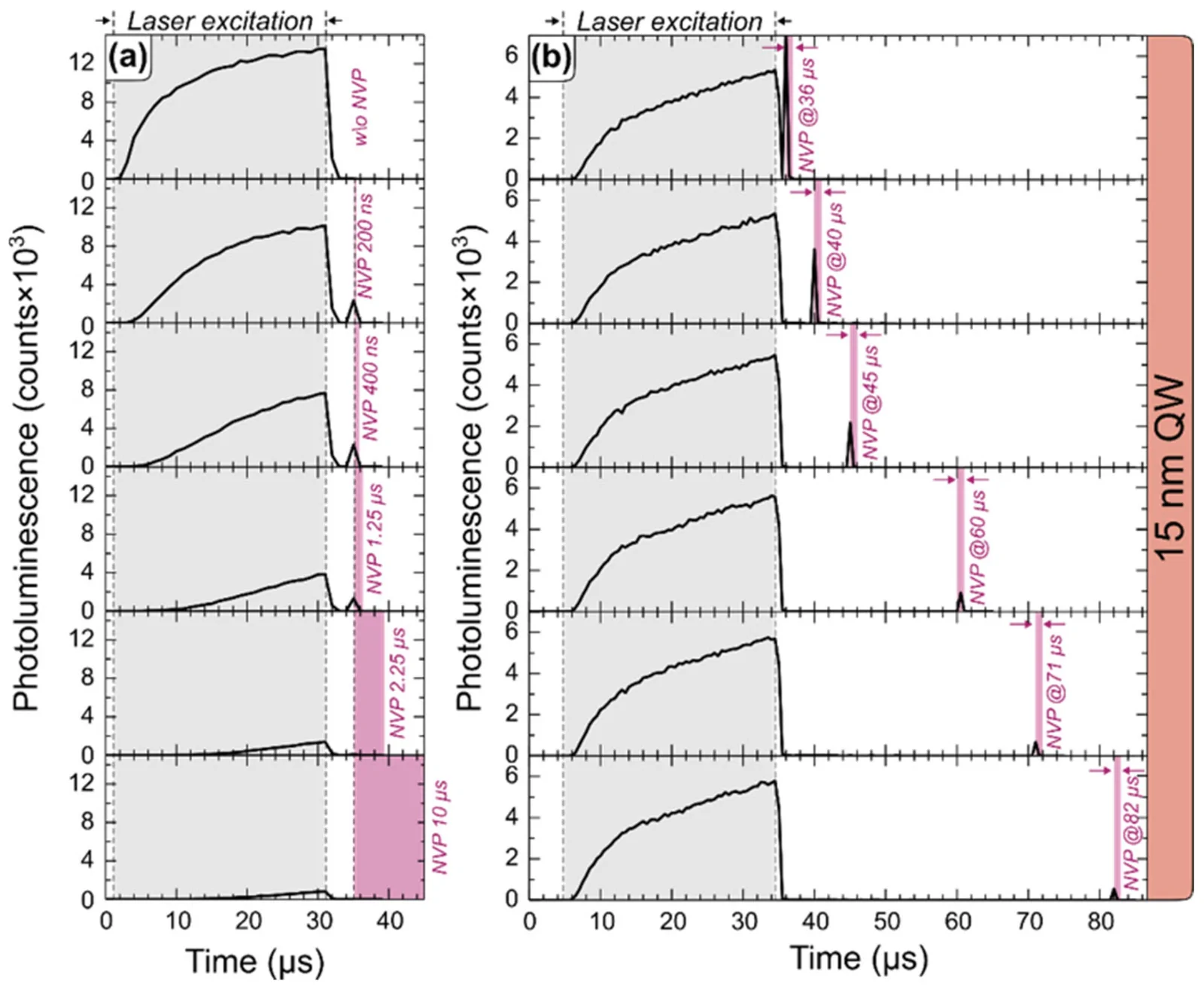
PL pulses excited by 3 mW, 30 μs pulses of the 405 nm LD (10% fill factor): (**a**) as a function of duration of the NVP applied 3 μs after the laser pulse (duration of the NVP increases from 0 to 10 μs); (**b**) as a function of delay of the 1 μs NVP with respect to the laser pulse (the NVP applied at increasing time delay after the excitation pulse) (reprinted with permission from [14]).

The NVPs applied after the laser pulse lead to the appearance of SPLs (short pulses of light), as discussed before. Those SPLs can be observed up to 100 μs after the laser pulse (Figure 13b). The SPLs are indeed short (10 ns), but the effect of NVPs on PL pulses persists up to NVPs duration of 10 μs or more (Figure 13a). After the application of negative voltage, the electric field is reduced in the well and some dark charge is “promoted” to excited states. This gives a short pulse of emission, followed by slower nonradiative decay of dark charge. In the following, it will be shown that the decay of dark charge during the NVP may be observed for milliseconds.

**Figure 14:**
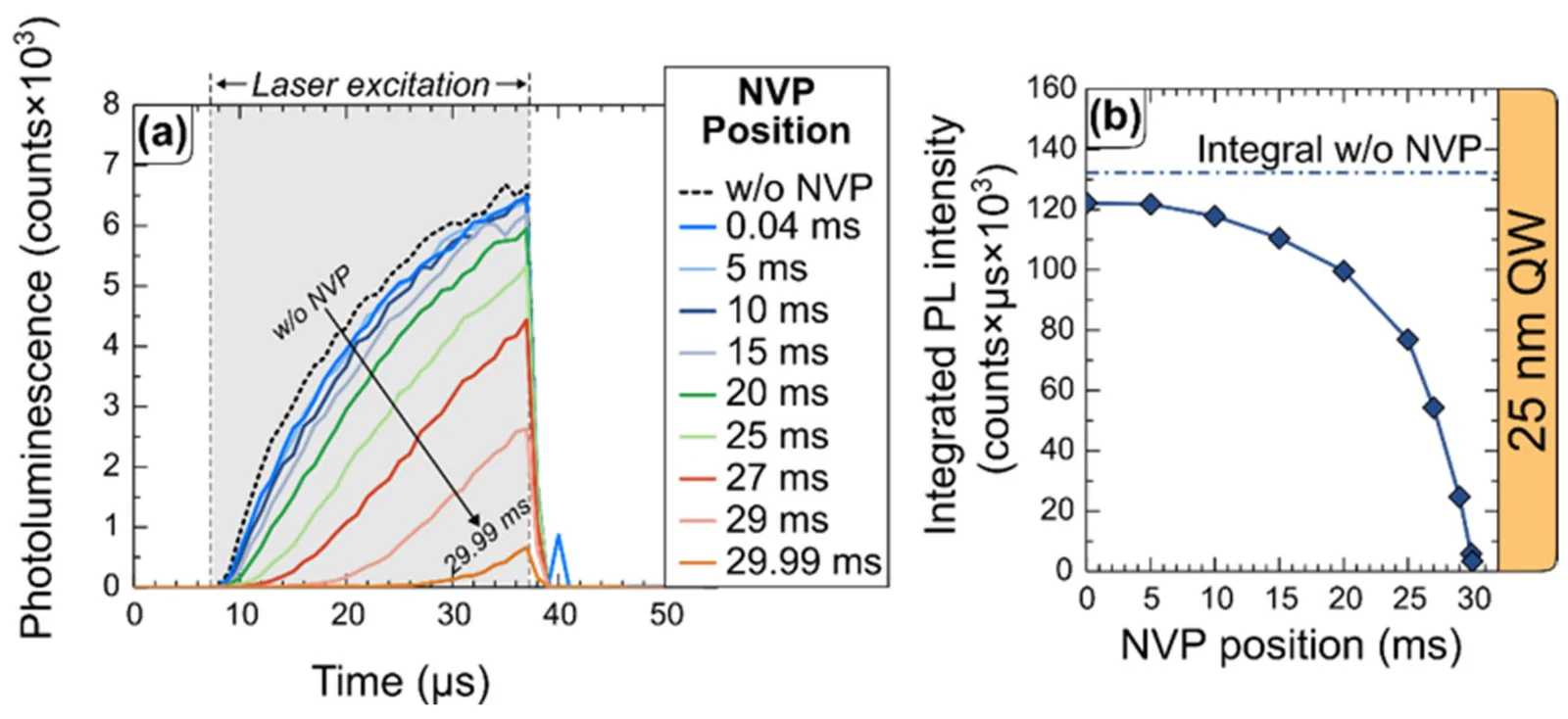
(**a**) PL pulses from the LED with a 25 nm well for different positions of the NVP (−10 V, 10 μs) relative to the laser pulse (given in the inset); the black dashed line shows the PL pulse without NVP. (**b**) Integrated PL intensity versus position of the NVP with respect to the laser pulse. The dashed–dotted line corresponds to the integrated PL intensity without NVP. The excitation was by 30 μs pulses of a 2 mW, 405 nm laser diode, and the excitation period was fixed at 30 ms (reprinted with permission from [14]).

Similar effects were observed for a 25 nm well. Another demonstration of the NVPs reducing the dark charge was obtained by fixing the excitation period at 30 ms and varying the position of the NVP (Figure 14).

**Figure 15:**
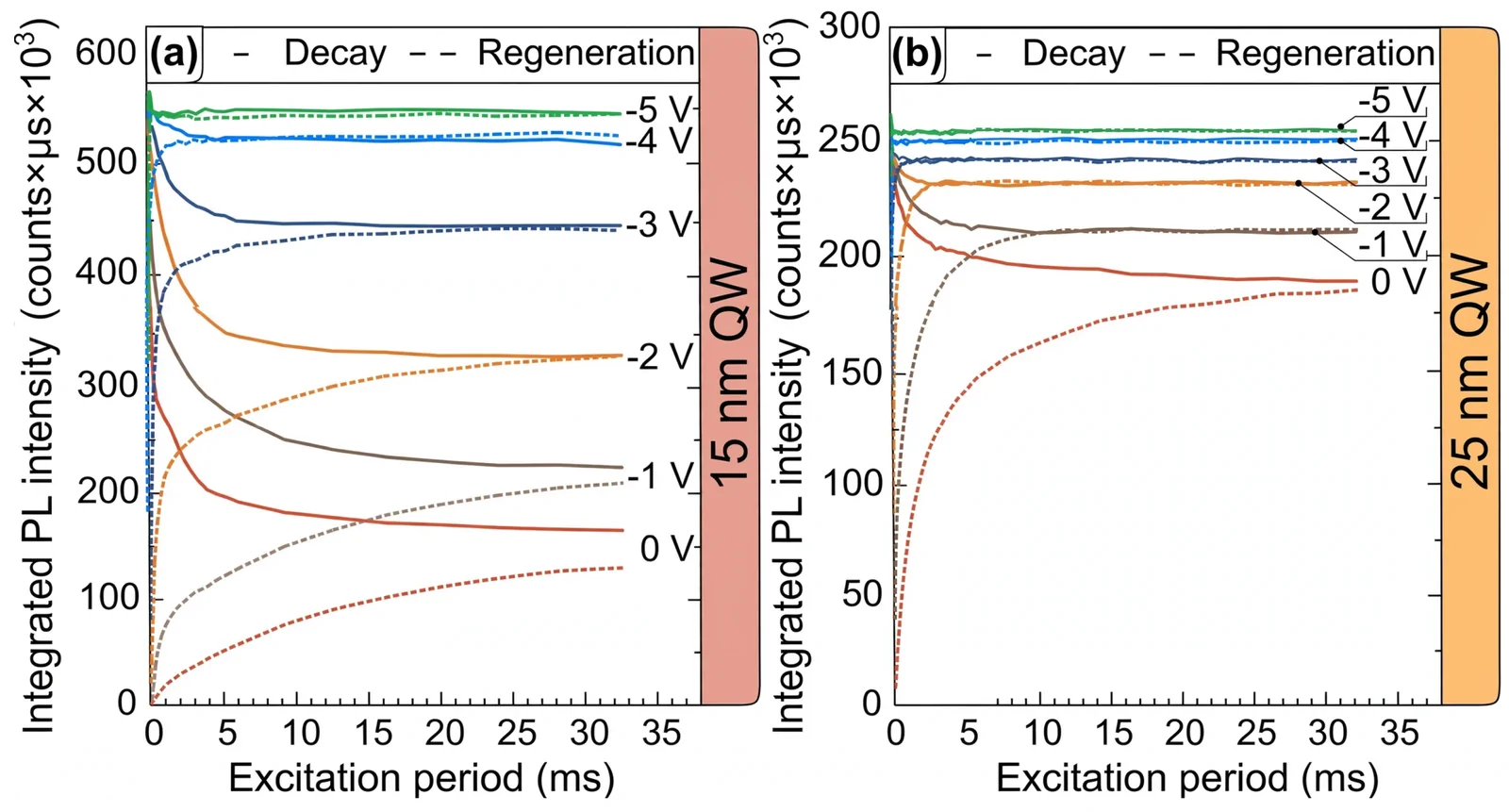
Integrated PL intensity for an LED with a 15 nm well (**a**) and for an LED with a 25 nm well (**b**) as a function of excitation period (for different CW voltages from 0 V down to −5 V). The excitation was with 405 nm, 3 mW, 30 μs laser pulses in (**a**) and 10 μs pulses in (**b**). Solid lines show the decay of PL pulses with increasing separation. Dashed lines show the regeneration of the PL signal after the application of the NVP with 10 μs duration and −10 V voltage. The NVP was applied 3 μs after the laser excitation pulse (reprinted with permission from [14]).

The data in Figure 12, Figure 13 and Figure 14 were obtained for the diode at V = 0 (closed circuit). When the reverse bias was applied to the diode, the PL pulses increased, but the effects of delay and regeneration (changes with excitation period) decreased (Figure 15). This is due to the fact that reverse bias lowers the field in the well and the effect of NVPs (switching to −10 V) is reduced.

**Figure 16:**
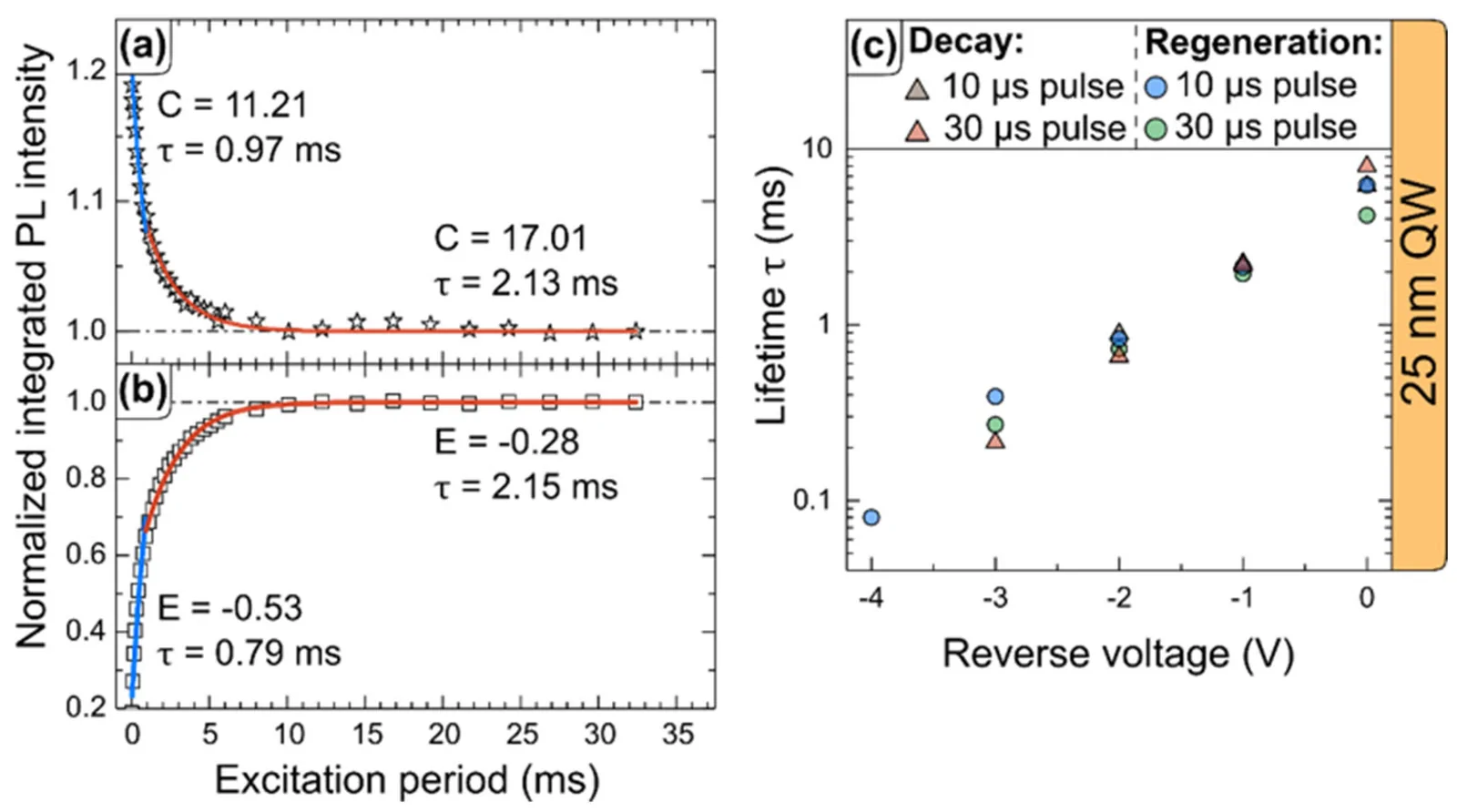
Examples of fits of PL decay (**a**) and regeneration (**b**) for an LED with a 25 nm well at V = −1 V. The data below 1 ms (fitted with a blue line) show fast decay/regeneration, while the data above 1 ms (fitted with a red line) show much slower variation. (**c**) Lifetime of “dark charge” vs. reverse voltage obtained from the fits for the 25 nm well (reprinted with permission from [14]).

A simple kinetic model with exponential decay and regeneration of dark charge was presented in [14]. However, since during the first 0.5–1 ms the decay/regeneration is fast and it slows down for longer times, it was necessary to fit the data in two regions: up to 1 ms and above 1 ms. The decay/regeneration times obtained from the fits (in the range above 1 ms) for the 25 nm well sample are shown in Figure 16c as a function of reverse voltage. The scatter of values for τ is large, but the strong reduction with voltage is clear. The decay and regeneration times have similar values.

These long decay/regeneration times of dark charge are due to very small overlaps of electron and hole wavefunctions. As shown in [18], the nonradiative recombination in nitride quantum wells through field-assisted multiphonon point defect recombination scales with the wavefunction overlap, similarly to radiative recombination. This is the main reason for the relatively high efficiency of nitride LEDs, (despite the strong built-in fields).

Such long lifetimes of dark charge may be attractive for charge-coupled or memory devices where information is stored in long-lived carrier populations.

### 4.4. Electroluminescence from Wide Wells in the Microsecond Range

**Figure 17:**
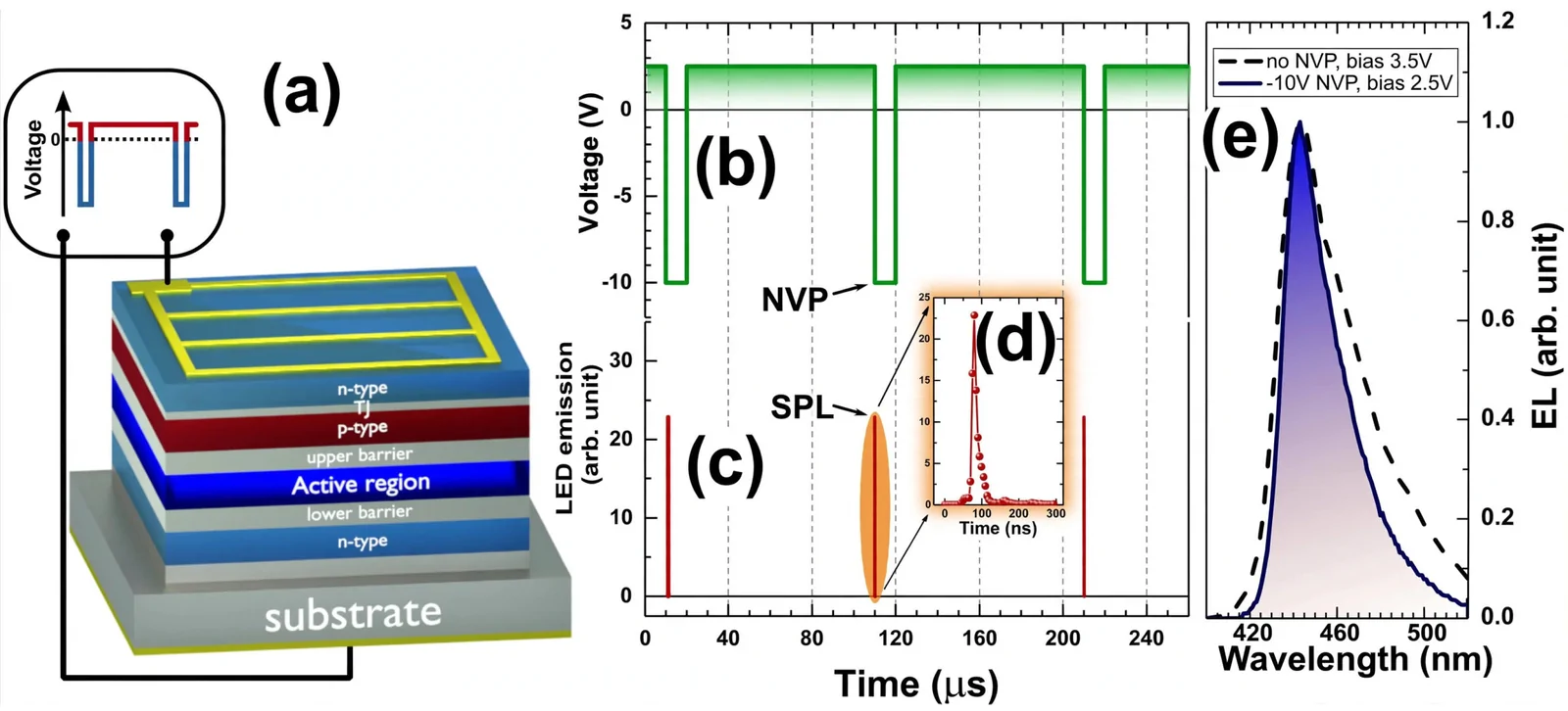
(**a**) Schematic structure of the LED, (**b**) voltage applied to the diode and (**c**) emission intensity from LED as a function of time (for forward voltage of +2.5 V and the period of NVPs equal to 100 μs), (**d**) magnified SPL intensity vs. time, and (**e**) normalized emission spectrum of SPL at −10 V (solid line) and the normalized electroluminescence spectrum of LED at 3.5 V (dashed line) (reprinted with permission from [15]).

Pulses of positive voltage (current) in the 800 ns range, followed by negative voltage, resulted in SPLs at the trailing edge of the pulse, as shown in [13]. The separation of the excitation pulses (and their duration) was fixed in these experiments. Using the photomultiplier for detection and a pulse generator for excitation, it was possible to perform a similar experiment using NVPs [15]. All parameters could be varied in a wide range: duration of NVPs, period of NVPs, and voltages. The experiment consisted of driving the diode with a low CW positive voltage (below the threshold for emission, typically 1–3 V) and periodically applying NVPs. Depending on the period of NVPs and on the value of the positive voltage, the diode started to emit SPLs at the beginning (leading edge) of each negative-voltage pulse. There was no emission in between the NVPs (Figure 17). This experiment is somewhat similar to the regeneration of PL in [14].

The NVPs deplete the QW of dark charge (partially or completely, depending on their voltage and duration). If their separation is short, dark charge does not recover sufficiently until the next NVP and the emission (SPL) is absent. If the separation of NVPs is long enough and the current pumping the well is sufficient, the emission appears at the beginning (leading edge) of each NVP.

**Figure 18:**
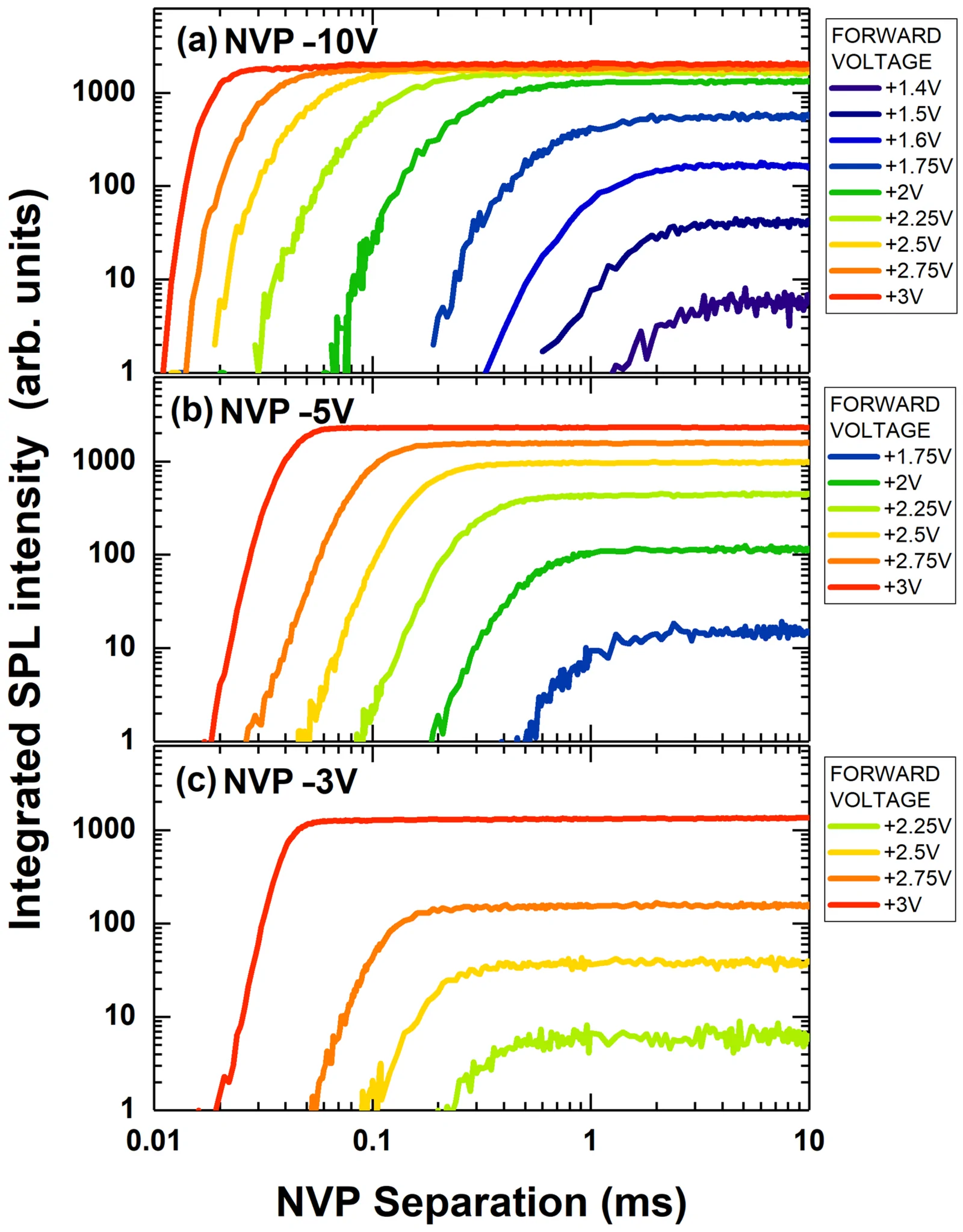
Integrated intensity of SPLs as a function of separation of 10 μs NVPs, for different forward voltages applied to the LED and three values of the NVP voltage: (**a**) −10 V, (**b**) −5 V, and (**c**) −3 V. SPLs appear at some threshold separations of NVPs and saturate. They increase for increased forward voltages (currents) between NVPs and for increased negative voltages during NVPs (reprinted with permission from [15]).

The dependence of the integrated intensity of SPLs on the positive voltage and on the period of NVPs is shown in Figure 18 (for the LED with 25 nm well). The separation between subsequent NVPs is increased (from 10 μs up to 10 ms) so that the charging time of the well is increased. The integrated intensity of SPLs is shown as a function of this separation (for several forward voltages), as shown in Figure 18a. For low voltages up to 1.25 V, there is no measurable emission from the sample. At 1.4 V the SPLs start to appear if the separation of NVPs (charging time) is above 1 ms. For increasing voltage (current), this “threshold separation” is reduced. Above the “threshold separation”, the intensity of SPLs increases and saturates. The saturation intensity of SPLs increases with forward voltage (current).

Similar experiments performed for lower NVPs voltages (−5 V and −3 V) showed lower intensity of SPLs and longer charging times required for their appearance (Figure 18b,c).

**Figure 19:**
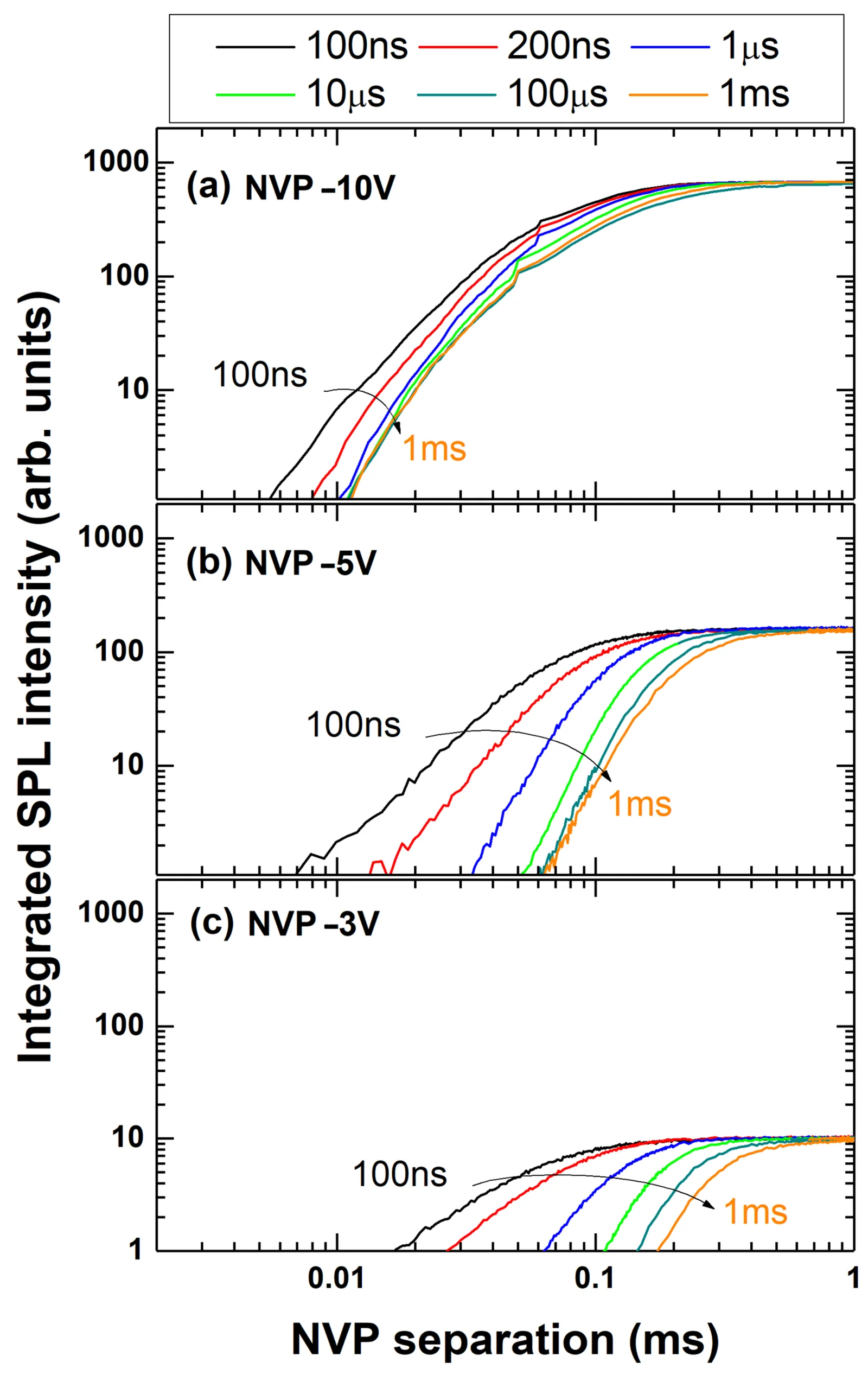
SPL intensity as a function of NVP separation (charging time) for three values of negative voltage applied during NVP—(**a**) −10 V, (**b**) −5 V, and (**c**) −3 V—and for increasing duration of the NVP (from 100 ns up to 1 ms). Here, the forward voltage applied to the LED was 2.5 V, and only the parameters of NVPs (voltage and duration) were varied. When NVP duration is long enough, dark charge is depleted during NVPs, and SPL intensity versus charging time stabilizes (reprinted with permission from [15]).

Increasing the duration of NVPs (from 100 ns up to 1 ms) requires longer charging times for the SPLs to appear (Figure 19). This means that during the NVP there is nonradiative recombination of dark charge for a much longer time than the duration of SPLs (10 ns). The duration of NVPs to achieve maximum depletion of dark charge in the well depends on the negative voltage of NVPs; for −10 V, it is about 1–10 μs; for −5 V, it is around 100 μs; and for −3 V, it is longer than 1 ms.

## 5. Numerical Simulation of Transient Electroluminescence

**Figure 20:**
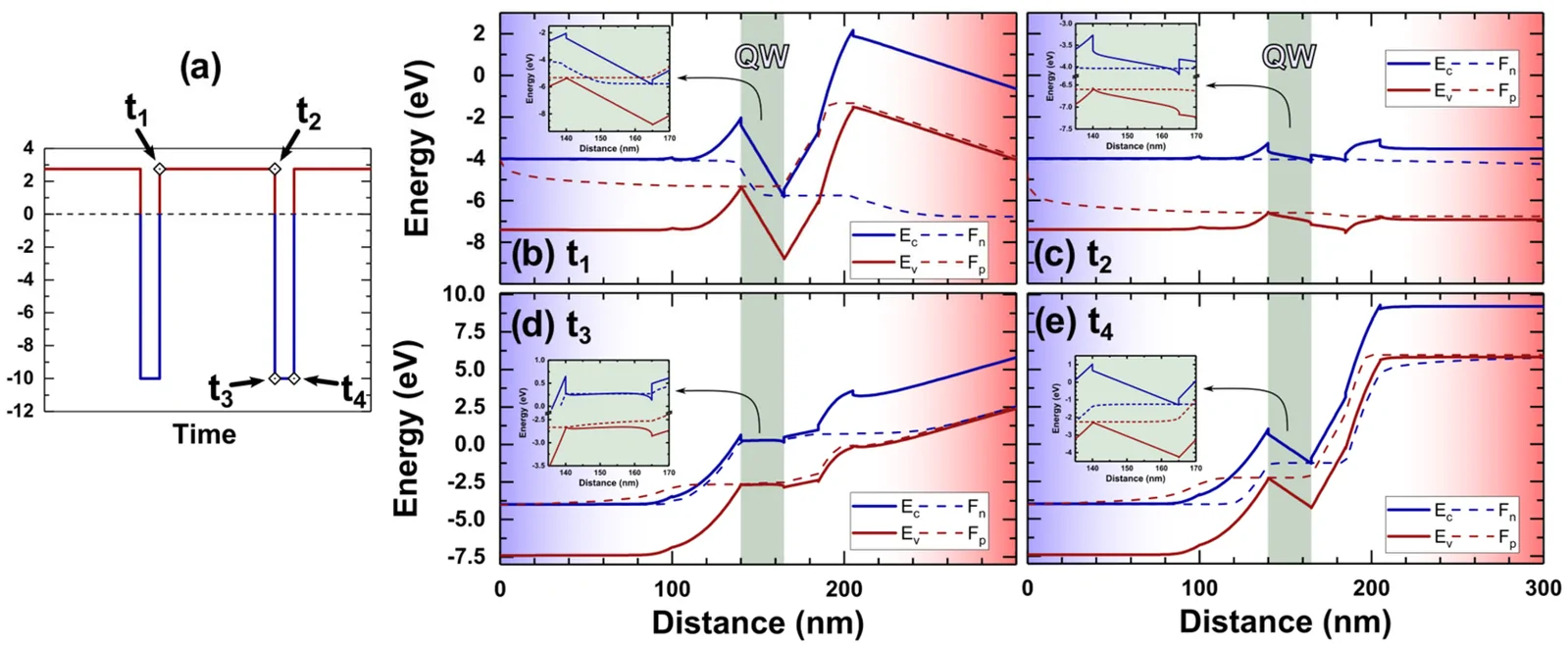
Band diagrams for an LED at four moments (t_1_, t_2_, t_3_, and t_4_) of the periodic experimental procedure (marked in (**a**)), starting from top left: (**b**) depleted well at 2.75 V, (**c**) charged well after 1.5 ms charging time at 2.75 V, (**d**) charged well just after application of an NVP at −10 V, (**e**) discharged well at the end of an NVP at −10 V. Quasi-Fermi levels are shown with dashed lines for electrons (blue) and for holes (red). Expanded band diagrams in the well region (together with electron and hole charge densities) are shown in the inserts (reprinted with permission from [15]).

In Ref. [15], a transient one-dimensional drift-diffusion model was used to simulate the time-dependent evolution of emission from a 25 nm well, subject to periodic NVPs. In Figure 20, the band profiles of the active layer of the diode are shown in four steps of the experimental procedure.

The simulation starts at t_1_, right after the NVP. The well is depleted of most of the dark charge; therefore, the field is large (Figure 20b). Between t_1_ and t_2_ (for 1.5 ms), the well is charged by low-intensity current and the field becomes partly screened, but the electrons and holes are still separated (Figure 20c). There is no emission from the well. When the voltage is rapidly lowered to −10 V, the field vanishes in most of the well, giving rise to a short (10 ns) pulse of light (SPL) at the beginning of the NVP. During the NVP (from t_3_ to t_4_), the concentration of dark charge is further reduced (through nonradiative SRH recombination) so that at moment t_4_ the field is nonzero.

**Figure 21:**
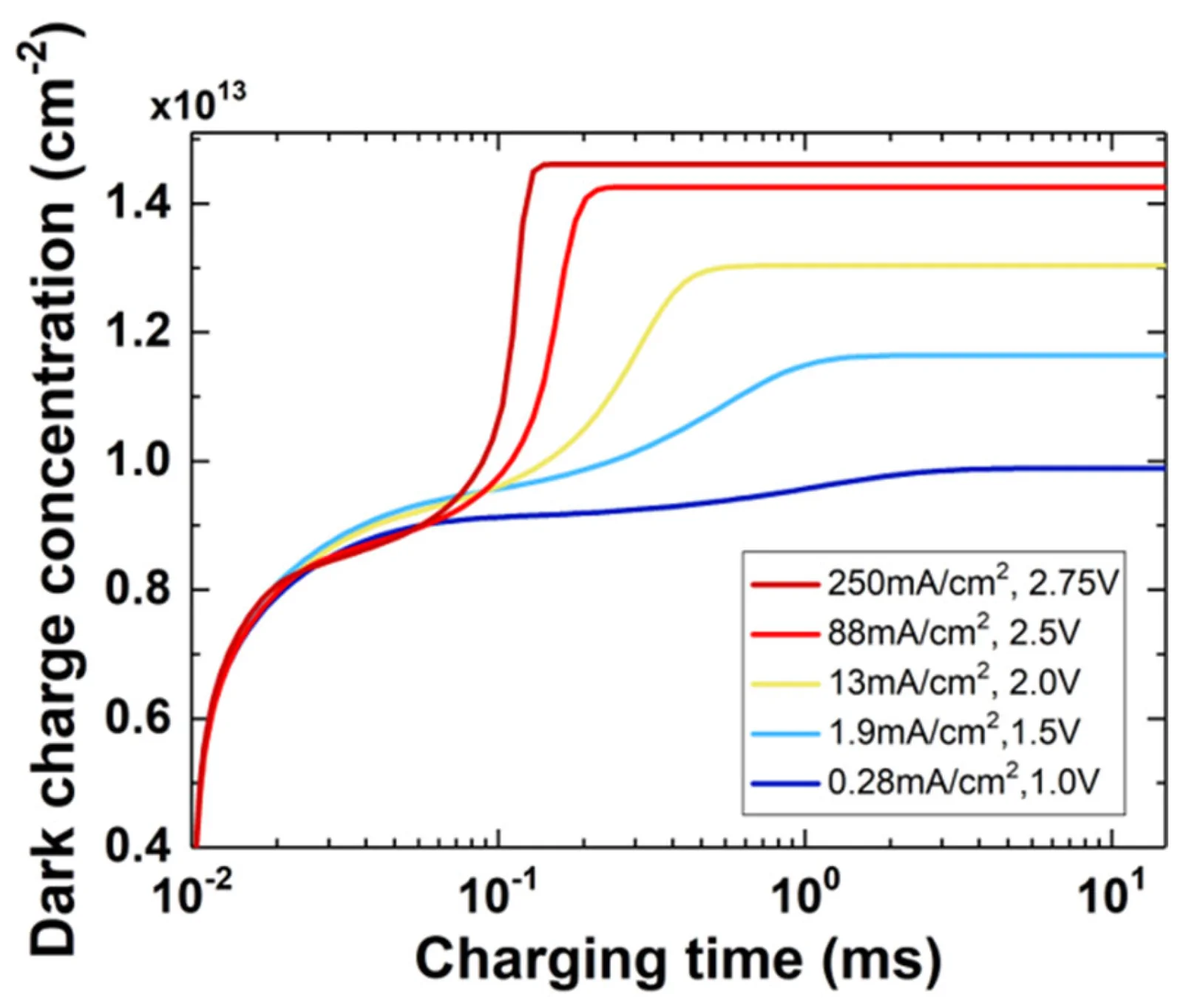
Concentration of electrons in the quantum well for different dc forward voltages applied to the LED (as indicated in the legend). This simulation starts right after the NVP at the t_1_ moment. Corresponding current densities (in mA/cm^2^) are also listed in the legend (reprinted with permission from [15]).

The accumulation of dark charge after the NVP (moment t_1_) under positive bias until the next NVP (moment t_2_) calculated in [15] is shown in Figure 21.

**Figure 22:**
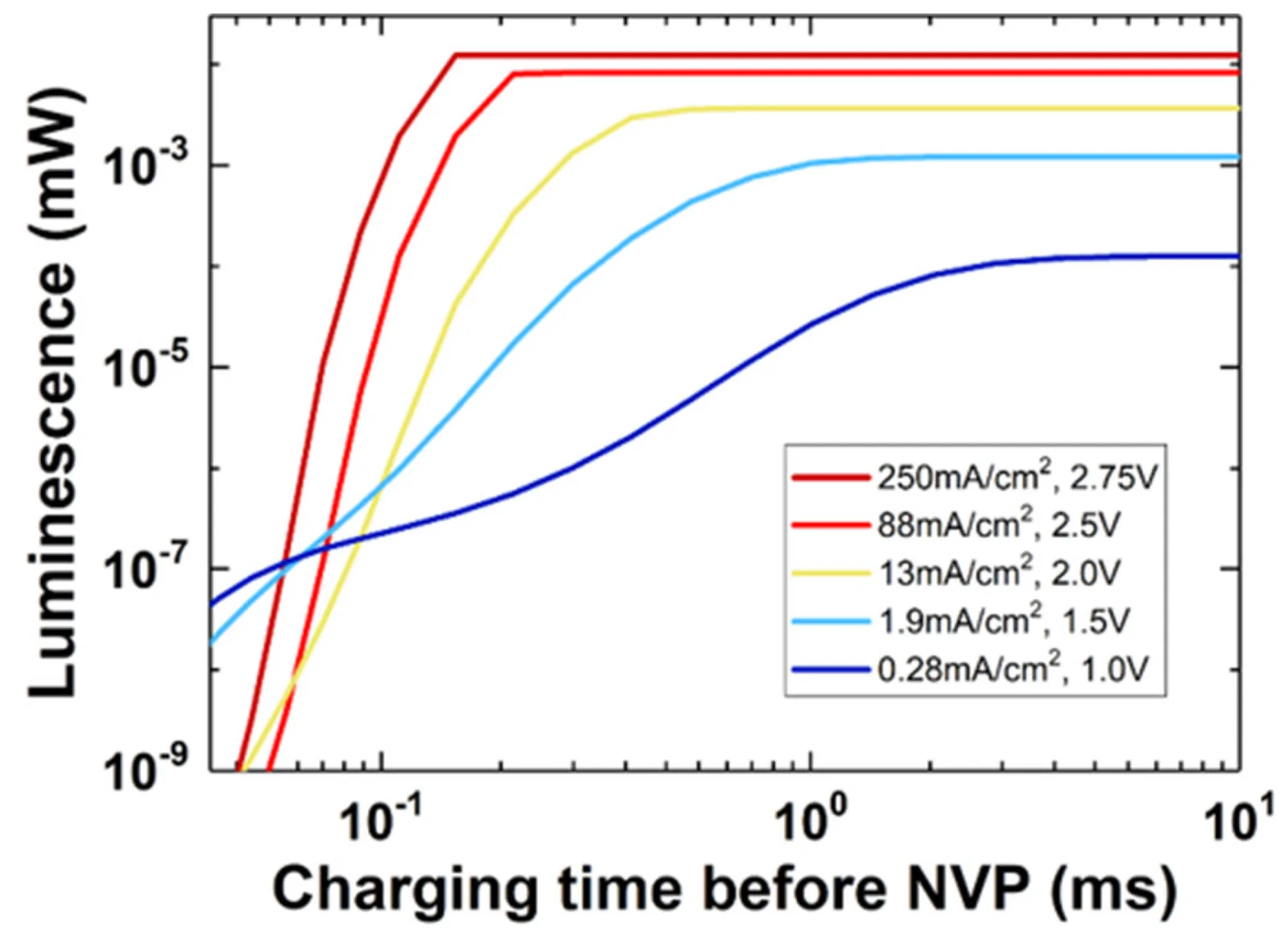
Simulated intensity of SPLs (generated by 10 μs, −10 V NVPs) as a function of charging time before the NVP, for different forward voltages applied to the LED (indicated in the legend, together with corresponding current densities in mA/cm^2^) (reprinted with permission from [15]).

Finally, the dependence of emission peaks (SPLs) on the period between NVPs (charging time) was calculated (Figure 22). The structure is initially set in the depleted-charge state (moment t_1_), and then the transient simulation starts with a low forward bias imposed, which is not sufficient to turn on the normal LED operation (from t_1_ to t_2_). After some charging time t_2_-t_1_, which is varied from 0.1 ms up to 10 ms, the −10 V NVP is applied for 10 µs, and then the forward bias is restored for a short time and the simulation ends. The results agree qualitatively with the experimental data, as shown in Figure 18. In theory, the emission is always present (though at extremely low levels), while in the experiment, it appears at some threshold level, depending on the sensitivity of the detector.

## 6. Summary and Conclusions

The transient properties of wide InGaN QWs in different time domains reveal some specific effects not encountered in regular, i.e., narrow, wells. In picosecond TRPL experiments, fast radiative recombination from hot-carrier plasma has been observed at low temperatures. The power dependence of emission showed a threshold behavior, attributed to the accumulation of dark charge. Electroluminescence in the nanosecond range showed a delay of emission with respect to the current pulse, decreasing with increasing current. Fast switching from positive to negative voltage revealed SPLs (with about 10 ns duration).

In these experiments, the period of excitation was fixed. In the experiments using a gated photomultiplier in the microsecond range, both PL and EL pulses have been studied as a function of their separation. The presence of dark charge caused a decay of PL pulses while increasing their separation up to 30 ms. The application of NVPs allowed us to decrease the amount of dark charge, generating SPLs at the beginning of the NVP and enabling slow recovery of the dark charge population until the next NVP.

Electroluminescence studies with the application of NVPs determined the conditions for the appearance of SPLs, in agreement with numerical simulation. The duration of NVPs up to 1 ms affected the appearance and intensity of SPLs, showing the slow recombination of dark charge due to nonradiative transitions (Shockley–Read–Hall). The possibility of achieving lasing during SPLs remains an open question.

The effective screening of the built-in field by dark charge (allowing for emission between excited states) and the stability of emission energy make wide-well LEDs and LDs worth further research. A long lifetime for dark charge could be interesting for charge-coupled or memory devices. And, above all, wide InGaN quantum wells provide new interesting physical effects.

## Figures and Tables

**Table 1:** Dependence of carrier density on current density obtained from the simulation in [16].

Current density (A/cm^2^)	0.91	2.8	9.06	49.1	221	1062	2023	4780
Carrier density (10^13^/cm^2^)	1.36	1.4	1.45	1.65	2.13	3.06	3.53	4.21

## Data Availability

No new data were created or analyzed in this study. Data sharing is not applicable to this article.

## References

[1] Bai J., Wang T., Sakai S. (2000). Influence of the quantum-well thickness on the radiative recombination of InGaN/GaN quantum well structures. J. Appl. Phys..

[2] Gardner N.F., Müller G.O., Shen Y.C., Chen G., Watanabe S., Götz W., Krames M.R. (2007). Blue-emitting InGaN–GaN double-heterostructure light-emitting diodes reaching maximum quantum efficiency above 200A∕cm^2^. Appl. Phys. Lett..

[3] Maier M., Köhler K., Kunzer M., Pletschen W., Wagner J. (2009). Reduced nonthermal rollover of wide-well GaInN light-emitting diodes. Appl. Phys. Lett..

[4] Laubsch A., Bergbauer W., Sabathil M., Strassburg M., Lugauer H., Peter M., Meyer T., Brüderl G., Wagner J., Linder N. (2009). Luminescence properties of thick InGaN quantum-wells. Phys. Status Solidi.

[5] Muzioł G., Hajdel M., Siekacz M., Turski H., Pieniak K., Bercha A., Trzeciakowski W., Kudrawiec R., Suski T., Skierbiszewski C. (2022). III-nitride optoelectronic devices containing wide quantum wells—Unexpectedly efficient light sources. Jpn. J. Appl. Phys..

[6] Bercha A., Trzeciakowski W., Muzioł G., Tomm J.W., Suski T. (2023). Evidence for “dark charge” from photoluminescence measurements in wide InGaN quantum wells. Opt. Express.

[7] Binks D.J., Dyer D., Fieldhouse-Allen W.R., Wallis D.J. (2026). Recent progress in cubic GaN epilayers and InGaN/GaN quantum wells. J. Phys. D Appl. Phys..

[8] Uždavinys T.K., Becerra D.L., Ivanov R., DenBaars S.P., Nakamura S., Speck J.S., Marcinkevičius S. (2017). Influence of well width fluctuations on recombination properties in semipolar InGaN quantum wells studied by time- and spatially-resolved near-field photoluminescence. Opt. Mater. Express.

[9] Marcinkevičius S., Kelchner K.M., Nakamura S., DenBaars S.P., Speck J.S. (2013). Optical properties of extended and localized states in *m*-plane InGaN quantum wells. Appl. Phys. Lett..

[10] Song J.-H., Kim T.-S., Park K.-N., Lee J.-G., Hong S.-K., Cho S.-R., Lee S., Cho M.W. (2014). Experimental verification of effects of barrier dopings on the internal electric fields and the band structure in InGaN/GaN light emitting diodes. Appl. Phys. Lett..

[11] Tomm J.W., Sakowski K., Bercha A., Muzioł G., Becht C., Schwarz U.T., Trzeciakowski W. (2025). Fast non-equilibrium carrier dynamics in polar InGaN/GaN structures with wide quantum wells. Appl. Phys. Lett..

[12] Uhlig L., Tepaß J., Hajdel M., Muzioł G., Schwarz U.T. (2023). Transition between quantum confinement and bulklike behavior in polar quantum wells. Phys. Rev. B.

[13] Tepaß J., Uhlig L., Hajdel M., Muzioł G., Schwarz U.T. (2023). Bright Emission at Reverse Bias After Trailing Edge of Driving Pulse in Wide InGaN Quantum Wells. Phys. Status Solidi A.

[14] Bercha A., Muzioł G., Chlipala M., Trzeciakowski W. (2023). Long-Lived Excitations in Wide (In,Ga)N/GaN Quantum Wells. Phys. Rev. Appl..

[15] Bercha A., Sakowski K., Muzioł G., Hajdel M., Trzeciakowski W. (2024). Light emission at reverse voltage in a wide-well (In,Ga)N/GaN light-emitting diode. Phys. Rev. Appl..

[16] Hajdel M., Gołyga K., Siekacz M., Feduniewicz-Żmuda A., Skierbiszewski C., Schwarz U.T., Muzioł G. (2025). Distinctness of Electroluminescence and Optical Gain in Laser Diodes with Wide Polar Quantum Wells. ACS Photonics.

[17] Murdin B.N., Heiss W., Langerak C.J.G.M., Lee S.-C., Galbraith I., Strasser G., Gornik E., Helm M., Pidgeon C.R. (1997). Direct observation of the LO phonon bottleneck in wide GaAs/AlxGa12xAs quantum wells. Phys. Rev. B.

[18] David A., Hurni C.A., Young N.G., Craven M.D. (2017). Field-assisted Shockley-Read-Hall recombinations in III-nitride quantum wells. Appl. Phys. Lett..

